# RNA-Seq uncovers endogenous NO-induced hormone signal transduction and carbon metabolism in response to PEG stress in alfalfa

**DOI:** 10.1186/s12864-025-11706-7

**Published:** 2025-05-23

**Authors:** Ying Zhao, Xiaofang Zhang, Yizhen Wang, Qian Ruan, Baoqiang Wang, Xiaoyue Wen, Xiaohong Wei

**Affiliations:** 1 College of Life Science and Technology, Gansu Agricultural University, Lanzhou, 730070 China; 2 Gansu Provincial Key Laboratory of Aridland Crop Science, Gansu Agricultural University, Lanzhou, 730070 China; 3 Gansu Key Laboratory of Crop Improvement & Germplasm Enhancement, Lanzhou, 730070 China

**Keywords:** *Medicago sativa*, Water deficit, Transcription regulation, Nitric oxide, TCA cycle

## Abstract

**Background:**

Alfalfa (*Medicago sativa* L.) has the benefits of high yield and nutritional value as a sustainable forage. However, the water deficit significantly limits its growth and yield performance. Nitric oxide (NO) is a signal molecule that can enhance plant tolerance. The majority of previous studies focus on the role of exogenous NO in plant tolerance. However, the underlying mechanism of endogenous NO in alfalfa drought tolerance remains largely unexplored.

**Results:**

To explore the mechanism of the endogenous NO-mediated water deficit resistance in the alfalfa, seedlings were exposed to polyethylene glycol 6000 (PEG) and NO scavenger (cPTIO). Results showed that PEG treatment significantly augmented alfalfa endogenous NO, MDA, O_2_^·−^, and H_2_O_2_ levels. In parallel, eliminating endogenous NO under PEG stress (PEG-NO) significantly diminished NO level, exacerbated MDA and reactive oxygen species accumulation, and decreased the activities of key enzymes involved in carbon fixation and TCA cycle, such as Rubisco, FBA, PDH, α-KGDH, and SDH, as well as reduced ABA and IAA content in alfalfa leaves. RNA-Seq and bioinformatics analysis suggested that endogenous NO-responsive DEGs primarily relate to carbon metabolism and hormone signal transduction. In further studies of these DEGs, we speculated that *GH3*, *SAUR*, *SnRK2*, and *ABF* genes and FBA, GAPDH, SBP, and *CS* are critical genes in response to endogenous NO under PEG stress.

**Conclusions:**

In summary, our study innovatively proposes a mechanism model of how endogenous NO enhances alfalfa tolerance to water deficiency at the physiological and molecular levels. The novel candidate genes can give genetic resources for the subsequent molecular-assisted breeding of drought-resistant alfalfa crops.

**Supplementary Information:**

The online version contains supplementary material available at 10.1186/s12864-025-11706-7.

## Introduction

Drought is a costly natural hazard that has far-reaching impacts on agriculture, ecosystem and socio-economic outcomes [1]. A systematic review of the physiology of plant responses to drought claimed that despite the moderate increase in global arable land, with a population rising, an additional 1 million ha will be needed to ensure food security. Furthermore, water demand for agriculture could double by 2050, while freshwater availability is projected to decrease by 50% due to climate change [2]. Consequently, planting drought-tolerant plants suitable for water-scarce regions is an essential and effective means to crack the drought problem. Alfalfa (*Medicago sativa* L.) is a widely adapted perennial leguminous herbage with high biomass production and nutritional value, making it a key component in animal husbandry feed protein. In addition, alfalfa is relatively drought tolerant due to its ability to absorb water from deeper soil layers through its extensive root system, which makes it one of the most popular varieties in arid and semi-arid regions where water resources are limited [3]. It has been suggested that mild drought stress may benefit forage quality as drought-stressed alfalfa will accelerate its shift to reproductive growth [4]. However, moderate to severe drought stress can seriously decrease alfalfa yield by up to 72% [5]. Hence, reducing drought’s impact on alfalfa production is one of the most important scientific problems that need to be solved urgently.

When alfalfa is exposed to drought stress, it actively maintains water balance in the body through morphological response, physiological and biochemical response, and gene expression regulation. The crop root system plays a vital role in the water cycle of the soil-plant-atmosphere continuum. As the soil water potential decreases, both the root volume and taproot length of alfalfa increase, leading to an expanded area of the root system in contact with the surrounding soil; this expansion facilitates the absorption and utilization of deep soil water [6–7]. On the other hand, alfalfa can reduce water loss by changing leaf morphology [8], epidermal wax content [9], or stomatal structure [10–11]. However, drought stress induces stomata closure or partial closure, largely reducing intercellular carbon dioxide concentration and sequencing-inhibited photosynthesis [12]. Moreover, alfalfa maintains osmotic balance by adaptively increasing the potential of osmotic substances such as soluble sugars, soluble proteins, proline, betaine, etc [13–15]. Besides, alfalfa leaves’ ABA and IAA contents accumulated significantly, while GA_3_ and ZR contents were decreased in response to drought stress [16–17]. The transmission of plant hormone signals can lead to changes in the expression of drought-responsive genes and various physiological responses [18–21]. Transcriptomic analysis further revealed that genes involved in phenylpropanoid biosynthesis, flavonoid biosynthesis, lignin wax biosynthesis, and starch and sucrose metabolism pathways are an important molecular basis for alfalfa drought resistance [22]. Transgenic studies have shown that the overexpression of *MtBGLU17*, *MsMYBH*, and *MsNTF2L* can improves the alfalfa antioxidant capacity and membrane stability [23–25]. Concurrently, gene regulation in reaction to drought often alters the profile of metabolites within the plant. Non-targeted metabolomics analyses revealed that the glycine, serine, and threonine and sphingolipid metabolism are associated with the alfalfa drought resistance [26].

Nitric oxide (NO) is an essential endogenous signaling molecule that exerts various physiological functions in plants, including seed germination, root growth, stomatal movement, photosynthesis, and cell transportation [27–28]. Importantly, NO regulates plant drought response by significantly increased concentrations under abiotic stress [29–31]. NO regulates the levels of cellular ROS and toxicity through two main pathways: enzymatic and nonenzymatic antioxidant enzymes [32]. In addition, the NO effect on drought resistance is related to the changes in phenolic acid and flavonoid compounds [33], fructan ascorbate and glutathione metabolism [34], and polyamine biosynthesis [35]. At the molecular level, NO has been found to improve the stability of genomic DNA in drought-stressed plants and positively regulated the transcription of stress-adaptation-related genes, i.e., *Cyt-G6PD* (*GPD5*, *G6PD6*, and *G6PD7*) in soybean roots [36].To date, most research has aimed to clarify the role of NO-mediated plant drought resistance using exogenous NO donors, such as sodium nitroprusside (SNP), S-nitroso-glutathione, and S-nitroso-N-acetyl-D-penicillamine [37]. However, it is worth noting that the rate of NO generation and the half-life of NO donors in the solution varied, and several NO-affected metabolomic changes induced by the NO donors were not comparable [38]. Our previous studies found that alfalfa responded to drought through different metabolic pathways after being treated with the NO-donor SNP or NO-scavenger at the germination stage [39–40]. Exogenous NO-responsive miRNAs specifically regulated metabolic pathways such as ascorbic acid, tyrosine metabolism, starch and sucrose metabolism, amino acid synthesis, ribosome, and protein transport in response to drought stress [41]. However, Ruan et al. found endogenous NO-responsive miRNAs are related to hormones (ABA, ETH, and JA) and phenylpropane metabolism, they can inhibit or degrade the expression of target genes after transcription [42]. Wei et al. reveled that Msa-miR166 in response to drought stress varies between leaves and roots, demonstrating tissue-specificity and spatiotemporal specificity [43]. Therefore, we hypothesize that there are undiscovered endogenous NO-dependent genes in alfalfa seedling leaves that could participate drought resistance.

Therefore, in this study, we combined RNA-Seq and physiological methods to explore the leaves of alfalfa plants responses to water-deficit (PEG-6000) and the lack of NO under PEG. We aimed to: (1) provide insight into the transcriptome response of alfalfa seedlings to drought stress and the change in the absence of endogenous NO. (2) uncover genes and pathways related to endogenous NO-induced stress tolerance in alfalfa, which provides valuable insight for alfalfa breeding, especially for improving yield and stress tolerance. Consistently, our results reveal that endogenous NO enhances the water-deficit tolerance of alfalfa by regulating carbon metabolism and hormone signal transduction pathways. *GH3*, *SAUR*, *SnRK2*, *ABF*, as well as *FBA*, *GAPDH*, *SBP* and *CS* may be potential candidate target genes for increasing drought resistance.

## Materials and methods

### Plant materials and growth conditions

*Medicago sativa* L. cultivar ‘Sanditi’ (NO-sensitive) was kindly provided by the Gansu Academy of Agricultural Sciences, Lanzhou, Gansu, China. The seeds were sown in plastic pots (140 × 122 × 95 mm) filled with a sterilized vermiculite and peat (1:2, v: v; 250 g) mixture; the pots were placed in a growth chamber for 5 weeks at 60% relative humidity, 25 °C, 500 µmol·m^− 2^·s^− 1^ photosynthetic photon flux density and a photoperiod of 16 h light/8 h dark. Then, 5-week-old seedlings were thinned out to keep 20 plants per pot and randomly divided into four groups for treatment, nine pots in each group. We adopt the Polyethylene glycol-6000 (PEG) was employed to simulate water stress, along with 2 - (4-carboxyphenyl) − 4, 4, 5, 5 - tetramethylimidazoline − 1 - oxyl − 3 - oxide potassium (cPTIO, NO scavenger) externally to quenched endogenous NO. The treatments were normal control (CK), 200 µM cPTIO (-NO), 10% PEG (PEG), and 10% PEG + 200 µM cPTIO (PEG-NO. Water stress groups irrigation 40 mL 10%PEG and endogenous NO quenched groups foliar smearing 5 mL 200 µM cPTIO (0.1% tween20 was utilized to prepare the cPTIO solution) each pot. The treatments were applied every 24 h for a total of seven times. On day 7 of treatment, leaf and root samples were collected from each treatment, then frozen in liquid nitrogen and stored at -80 °C for RNA-Seq, qRT‒PCR, and physiological analysis. Three biological replicates were given in the experiments.

### RNA isolation and transcriptome sequencing

Total RNA was extracted from alfalfa leaf samples using TRIzol reagent (Invitrogen, USA). After validation of RNA quality, concentration, and integrity, a Nanodrop spectrophotometer (IMPLEN, CA, USA), Qubit 2.0 Fluorometer (Life Technologies, CA, USA), and Agilent 2100 system (Agilent Technologies, CA, USA) were used respectively. Then, samples were used to construct a transcriptome sequencing library following the manual of the NEBNext^®^Ultra™ RNA Library Prep Kit for Illumina^®^(NEB, USA), and then sequenced on an Illumina HiSeq 2000 system, and paired-end reads were generated.

### De Novo assembly, mapping and annotation

Raw data were cleaned by removing reads containing adapters, ploy-N and low-quality reads. The clean reads were mapped to the assembled transcriptome performed using the Trinity assembler [44], and then the mapped reads were used for all the downstream analyses. The unigene functions were annotated based on NR (NCBI non-redundant protein sequences), Swiss-Prot (a manually annotated and reviewed protein sequence database), KOG/COG/eggNOG (Clusters of Orthologous Groups of proteins), GO (Gene Ontology) and KEGG (Kyoto Encyclopedia of Genes and Genomes) using BLAST software [45]. After predicting the amino acid sequences of the unigenes, annotation formation was performed by searching against the Pfam (Protein family) database using HMMER software [46].

### Differential expression analysis

Differentially expressed genes (DEGs) in CK vs. PEG and PEG vs. PEG-NO comparative groups were identified using the DESeq R package [47]. The resulting P values were adjusted using Benjamini and Hochberg’s approach for controlling the false discovery rate (FDR). Genes with an FDR < 0.05 and|log_2_ fold change| ≥ 2 as differentially expressed.

### Validation of DEGs by qRT‒PCR

Total RNA samples from alfalfa leaves were isolated using an RNA simple total RNA kit (Tiangen, Beijing, China) according to the manufacturer’s instructions. PDF2 was used as an internal standard to calculate relative gene expression levels [48], and Primer Premier 6.0 was used to design the primer sequences (Table S1). cDNA synthesis and qRT-PCR analysis were performed using a one-step SYBR Prime Script PLUS RT-PCR kit (TaKaRa, Dalian, China). PCR amplification was performed in a 96-well platform (FTC-3000, Canada) according to the following protocol: one cycle at 42 °C for 5 min and 95 °C for 10 s, followed by 50 cycles at 95 °C for 5 s and 60 °C for 31 s. Melting curve analysis was performed after incubation at 60 °C for 34 s. The expression level of the candidate genes was calculated with the 2^−∆∆Ct^ method [49].

### NO detection and growth index measurement

The NO content was determined using the Total Nitric Oxide Assay kit (Kemin, Suzhou, China). Leaf or root material 0.1 g was accurately weighed. Then, we added 1 mL of precooled sodium phosphate (50 mmol/L, pH 7.0) buffer to grind the homogenate in an ice bath and centrifuged it at 10,000× g at 4 °C for 15 min. The supernatant was taken for NO determination with a spectrophotometer at wavelengths of 550 nm, and the determination was repeated 3 times. Seven-day-treatment seedlings were photographed for stem and root length measurement using ImageJ software. Thirty seedlings were measured for each line. Each treatment group had three biological replicates.

### Oxidative stress indicator (MDA, O_2_^·-^, H_2_O_2,_ ·OH) determination

The malondialdehyde content was measured using the thiobarbituric acid (TBA) method [50]. Approximately 0.3 g of fresh leaves were homogenized, centrifuged for 10 min at 12,000 × g, and then placed in 5 ml trichloroacetic acids (10%). The mixture containing 2 ml of supernatant and 2 ml of TBA (0.5%) was put into boiling water for 15 min, then quickly cooled and centrifuged at 12,000 × g for 10 min. The absorbance of the supernatant was measured at 450, 532, and 600 nm via a spectrometer.

The superoxide anion (O_2_^•−^) production rate was measured using the Elstner and Heupel method [51]. About 0.2 g of fresh leaves were homogenized in 2 mL ice-cold 100 mM phosphate buffer (pH 7.8). The homogenates were centrifuged at 12,000 × g for 30 min at 4 °C, and the supernatants were used to analyze the O_2_^·−^ production rate. The supernatant was added to 10 mM hydroxylamine hydrochloride at 25 °C for 1 h, 17 mM paminophenic acid, and 7 mM α-naphthylamine were added, then the absorbance at 530 nm after 20 min.

Hydrogen peroxide (H_2_O_2_) was also measured using Loreto and Velikova’s method [52]. About 0.3 g of fresh leaves were pulverized in a pestle and mortar containing 3 ml of trichloroacetic acid (1% w/v). After that, the extract was centrifuged at 15,000 × g for 15 min, and 0.75 ml of the filtrate was reacted with 0.75 ml of 10 mM K buffer and 1.5 ml of 1 M KI. The OD of all treated samples was recorded at 390 nm, and results were expressed as µmol H_2_O_2_ /g fresh weight.

The scavenging rate of hydroxyl radicals (•OH) was determined using hydroxyl free radical scavenging determination kits (Fenton method) provided by Beijing Solarbio Science & Technology (Beijing, China). Approximately 0.1 g of leaf tissue was added to 1 mL of extraction solution to homogenize in an ice bath and centrifuged at 10,000 g at 4 °C for 10 min. The supernatant was used for subsequent according to the procedures specified by the manufacturers. Each experiment was performed in triplicate, and each assay was performed three times.

### Carbon metabolism enzymes and amino acids assays

The key enzymes involved in carbon fixation include ribulose-bisphosphate carboxylase (Rubisco), glyceraldehyde-3-phosphate dehydrogenase (GAPDH), fructose-bisphosphate aldolase (FBA), and pyruvate dehydrogenase (PDH). The key enzymes involved in the TCA cycle include citrate synthase (CS), isocitrate dehydrogenase (ICD), and α- oxoglutarate dehydrogenase (α-KGDH), succinate dehydrogenase (SDH), and malate dehydrogenase (MDH). Carbon metabolites include citrate (CA) and amino acid (AA), and were measured using corresponding assay kits from Solarbio Life Sciences (Beijing, China) according to the manufacturer’s protocols. Leaf tissue weighed 0.1 g was added to 1 mL extraction solution and thoroughly homogenized in an ice bath and centrifuged at 4 ℃. Then, the supernatant was analyzed by spectrophotometry. Rubisco, GAPDH, FBA, ICD, MDH, and α-KGDH activities were detected at 340 nm in redox reaction assays. PDH, SDH and CS activities were determined at 605, 600 and 412 nm, respectively. The CA and AA contents were determined at 545 and 570 nm, respectively. All tests were performed in triplicate.

### ABA and IAA content detection and analysis

ABA and IAA were assayed as described by Qi et al. with some modifications [53]. Briefly, 1 g (fresh weight) of alfalfa leaves or roots for each treatment was quickly frozen in liquid nitrogen and ground into a fine powder and then transferred into a test tube. After adding 10 mL methanol/formic acid (99/1, v/v), samples were stored in a refrigerator at 4 ℃ for 24 h. Then, the supernatants were collected after centrifugation at 10,000 rpm for 10 min at 4 ℃. Subsequently, 4 mL ultrapure water was added to 1 mL supernatants to each sample, passed through the HC-C18 column, and then washed with 10% methanol and methanol-formic acid solution, separately. Finally, the eluate was collected and dried by nitrogen and then dissolved in 1.0 mL formic acid solution. Finally, the extract was filtered with a 0.22 μm filter for liquid chromatography-mass spectrometry (Agilent 1260–6460 LC/MS) detection. The chromatography and mass spectrometry conditions are presented in Mass spectrometry and were performed under negative ESI mode with multiple reaction monitoring (MRM) scan mode. The mobile phase was chromatographic methanol (A) and 0.1% acid (B). The flow velocity was 0.3 mL· min^− 1^, the column temperature was 30 ℃, and the injection volume was 5 µL. The sample ABA and IAA contents were calculated according to the standard curves. Multiple technical repetitions were performed using separately harvested material from different plants within the same experiment.

### Data statistics and analysis methods

The physiological and biochemical indices were analyzed separately and subjected to analysis of variance using (ANOVA) SPSS Inc. version 26.0. The significant differences between the treatment means were compared using the highest significant difference as obtained by the Tukey test at *P*<0.05 levels. Graphs were generated using OriginPro 2021 (Origin Software, USA).

## Results

### NO concentration and the morphology of the alfalfa seedlings

**Fig. 1:**
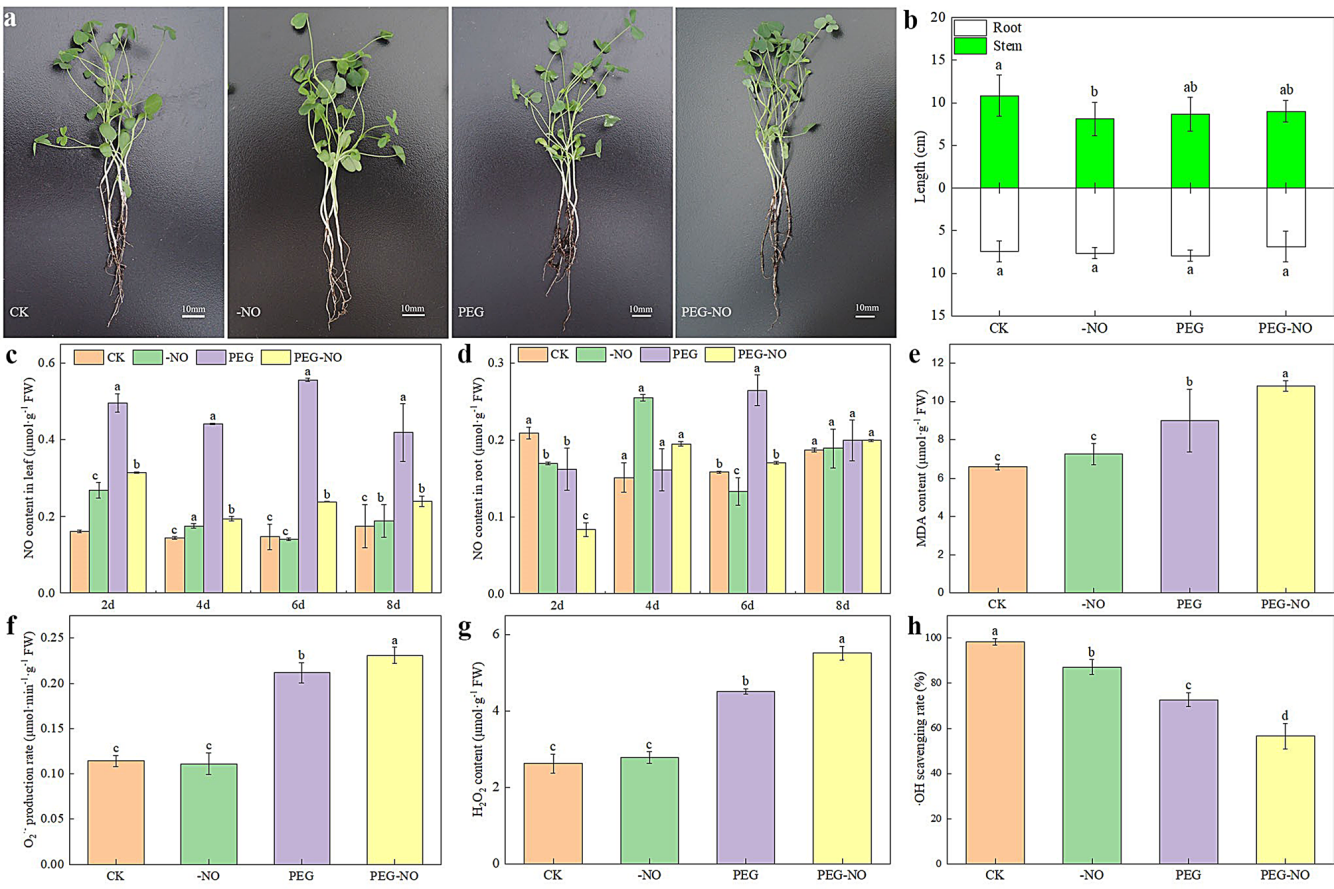
Changes of morphology and endogenous NO levels of alfalfa under treatments. (**a**) Photographs were taken after 7 days of different treatments. (**b**) Root and steam length. (**c**) NO content in leaf. (**d**) NO content in root. (**e**) MDA content. (**f**) O_2_^•−^ production rate. (**g**) H_2_O_2_ content. (**h**). •OH scavenging rate. CK, control; -NO, 200 µM cPTIO; PEG, -0.3 MPa PEG-6000; PEG-NO, -0.3 MPa PEG-6000 + 200µM cPTIO. Data are means ± SD calculated from three biological replicates. Lowercase letters indicate significant differences at 0.05 level by the Tukey test

The morphology of alfalfa seedlings under different treatments after six days is shown in Fig. 1a. Stem length was reduced by 19.85% under PEG treatment as compared to control. NO scavengers further inhibited stem elongation, while root length showed no differences between them (Fig. 1b). In addition, compared with CK, PEG treatment had no significant effect on the lateral roots number, PEG-NO treatment significantly decreased the lateral roots number, but PEG treatment significantly decreased the stem branches number, and there was no significant difference between PEG and PEG-NO (Fig.S1). Besides, the stomatal length, width, and conductance of the leaves were the lowest under PEG treatment, but these indicators of the PEG-NO treatment increased by 15.76%, 80.15% and 58.33% respectively (Fig.S2). When exposed to PEG stress, the production of endogenous NO in seedling leaves was significantly increased and peaked at 6d compared to the CK. Still, the PEG-NO treatment resulted in a significant decrease in NO levels compared to the PEG treatment (Fig. 1c). In contrast, NO levels in roots were generally lower than in leaves. A significant increase in NO content over the control was not observed until the sixth day after PEG treatment (Fig. 1d). These suggest that leaves are more sensitive to PEG stress than roots because NO accumulates quickly in leaves. Furthermore, we analyzed the physiological characteristics of alfalfa leaves after six days of treatment; MDA content, O_2_^•−^ production rate, and H_2_O_2_ content were significantly increased under PEG stress, and PEG-NO treatment further intensified oxidative damage (Fig. 1e, f, g). However, PEG treatment exhibited a lower •OH scavenging rate compared to CK, and PEG-NO treatment further reduced this effect (Fig. 1h). These results provide a theoretical basis for choosing alfalfa leaves treated for 6 days as the transcriptome sequencing sample.

### Transcriptome-scale analysis of NO-responsive genes

Illumina sequencing analysis was performed on leaves treated with PEG and PEG-NO, as well as CK and -NO (normal watered) to elucidate the molecular mechanism of endogenous NO in alfalfa drought tolerance. After data processing, a total of 31.05 (CK), 25.74 (-NO), 25.74 (PEG), and 27.16 million (PEG-NO) clean reads were generated, and approximately 71.94–75.33% of reads were mapped to the de novo assembled *Medicago Sativa* transcriptome (Table S2). Based on the gene expression information, a principal component analysis (PCA) of all samples shows PEG and NO scavenger-induced changes in alfalfa genes (Fig.S3a). Pearson’s correlation coefficients were used to evaluate relationships between biological replicates. The correlations between replicates ranged from 0.93 to 0.98, and the correlations between different treatments were between 0.25 and 0.73, indicating that the results were highly repeatable (Fig.S3b).

A total of 96,255 transcripts with a mean length of 902.94 bp and an N50 value of 1577 were obtained; the number of genes that could be mapped onto six public databases is shown in Table S3. Overall, the unigene sequences were most similar to gene sequences from *Medicago truncatula* (74.20%) within the NR database (Fig.S4). A total of 28,029 unigenes were classified into 55 functional groups with three subcategories, including biological processes (19), cellular components (16), and molecular functions (20) using GO classification (Fig.S5). Specifically, 24,483 unigenes were significantly homology to 25 KOG functional categories (Fig.S6).

### Identification and functional classification of DEGs

**Fig. 2:**
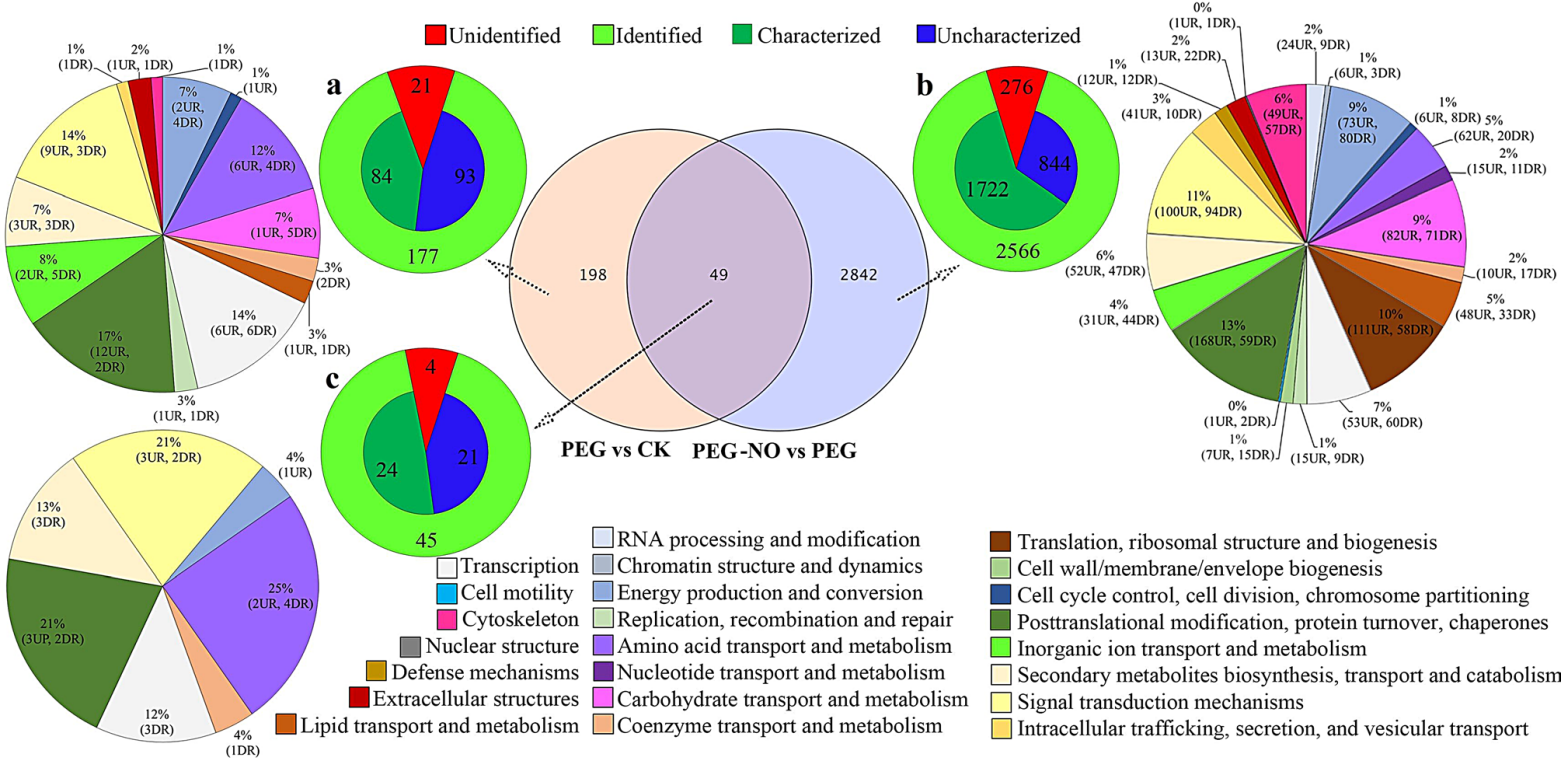
Distribution and functional classification of differentially expressed genes (DEGs) in PEG and PEG-NO treatment. (**a**) specifically DEGs under PEG stress; (**b**) specifically DEGs under NO scavenger stress; (**c**) common DEGs under PEG and PEG-NO. UR, up-regulated; DR, down-regulated

From the 96,255 transcripts, a total of 247(139 up-regulated, 108 down-regulated) and 2891(1595 up-regulated, 1296 down-regulated) DEGs were identified and analyzed between PEG vs. CK, and PEG-NO vs. PEG, respectively. The list of DEGs was provided in supplementary Table S4. For DEGs specifically in PEG and PEG-NO conditions, 21 and 276 DEGs corresponding showed no match information among SwissProt, Pfam, KOG, and NR databases. Of the 177 and 2566 identified DEGs separately in PEG and PEG-NO conditions, 93 and 844 DEGs yet defined function, and 84 and 1722 DEGs were matched with genes of known function, respectively. For the 1722 DEGs, including 980 up-regulated genes and 739 down-regulated genes. Based on the function description, the characterized DEGs were classified into 23 groups (Fig. 2). To further understand the mechanistic differences between the two comparations, DEGs categorized in clusters and groups were functionally annotated using the KEGG pathway database. We found DEGs in PEG treatment were significantly enriched in Circadian rhythm-plant and flavonoid biosynthesis pathways, and higher rich factors were observed. However, we identified DEGs in PEG-NO vs. PEG were mapped to the pathways including Carbon fixation in photosynthetic organisms, Oxidative phosphorylation, Carbon metabolism, Citrate cycle (TCA cycle), as well as plant hormone signal transduction (Fig.S7).

### Expression of the transcription factor in response to PEG and NO scavenger treatment

**Fig. 3:**
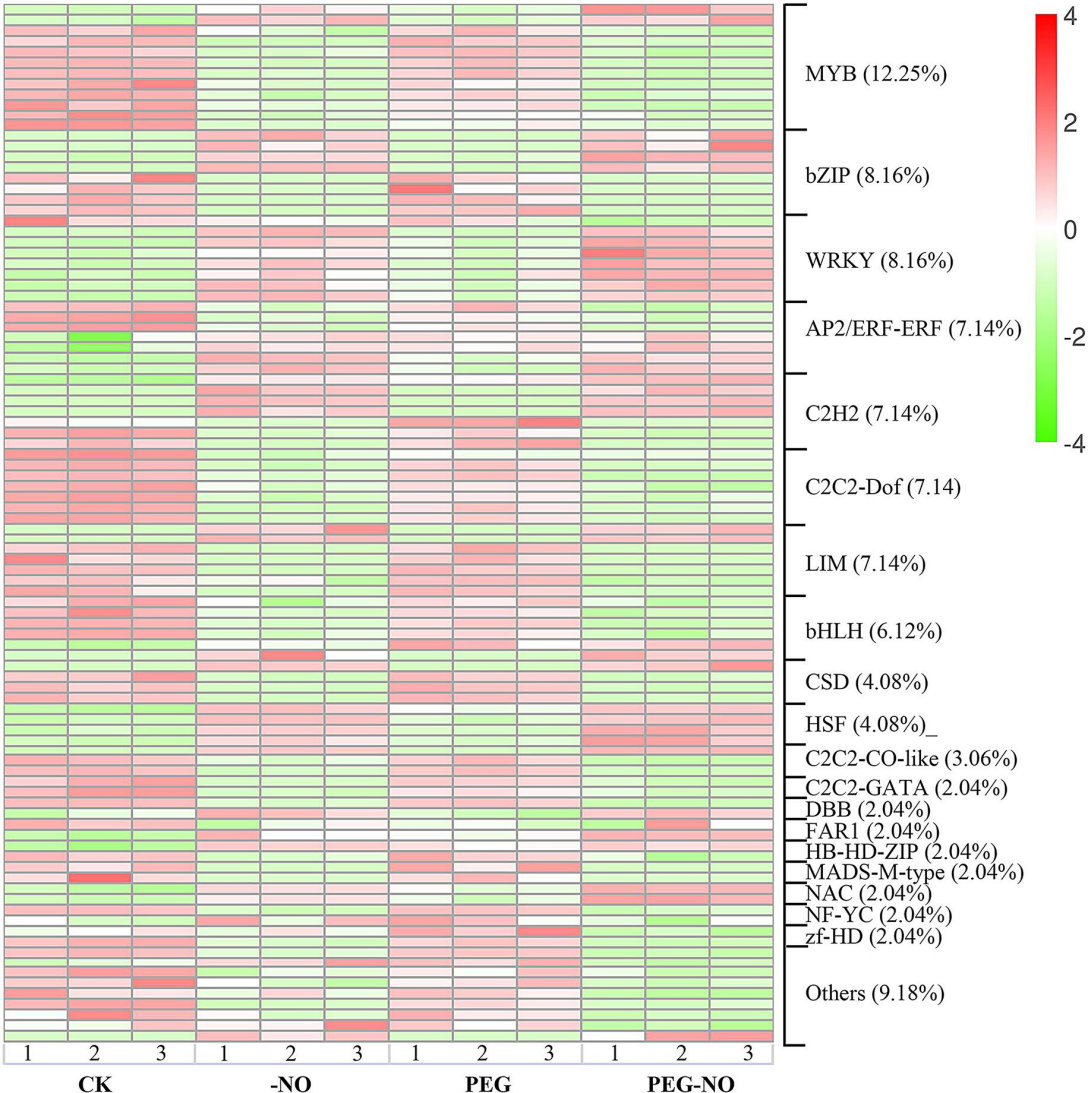
Heatmap of differentially expressed transcription factors in the control and stress treatments. Expression values of genes are presented as FPKM-normalized log_2_ -transformed counts. Red and blue colors indicate up- and down-regulated transcripts, respectively

Next, the DEGs encoding TFs were analyzed. A total of 98 DEGs encoding TFs were identified in alfalfa seedling leaves in response to drought stress and NO, and these TFs belonged to 24 TF families. Most of the identified DEGs encoded members of the MYB (12), bZIP (8), WRKY (8), AP2/ERF (7), C2H2(7), C2H2-Dof (7), LIM(7), bHLH (6), CSD (4), HSF(4) related TF families (Fig. 3, Table S5). The analysis of the TFs expression level by heatmap revealed a clear separation of all treated samples. Most of the TFs have similar expression patterns between CK and PEG; on the contrary, they have a distinguishingly expressed level under PEG-NO treatment, such as MYB, C2C2-Dof, and other TFs were typically down-regulated (Fig. 3). The TFs had different expression patterns in alfalfa seedling leaf responses to PEG, and PEG-NO, which suggested that endogenous NO involved drought stress resistance mechanisms.

### TCA cycle pathway and key metabolic enzymes

**Fig. 4:**
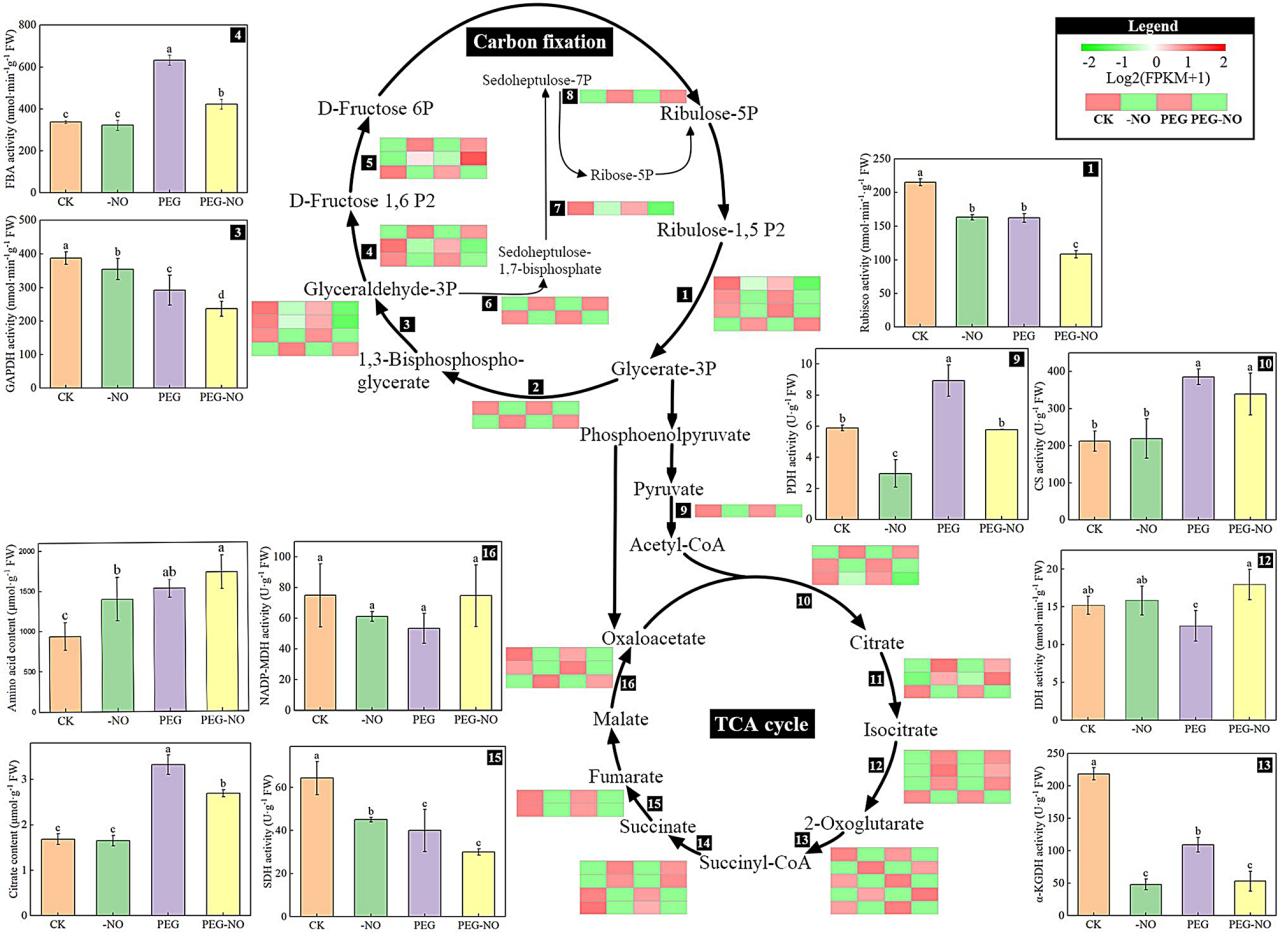
Schematic diagram of carbon fixation and TCA cycle metabolic pathway responses to PEG and NO stress in Medicago sativa leaves. Transcripts encoding different enzymes are represented in black boxes. 1: ribulose-bisphosphate carboxylase (Ru-bisco); 2: phosphoglycerate kinase (PGK); 3: glyceraldehyde-3-phosphate dehydrogenase (GAPDH); 4: fructose-bisphosphate aldolase (FBA); 5: fructose-1,6-bisphosphatase (FBP); 6: triosephosphate isomerase (TPI); 7: sedoheptulose-bisphosphatase (SBP); 8: transketolase (TKT); 9: pyruvate dehydrogenase (PDH); 10: citrate synthase (CS); 11: aconitate hydratase (ACO); 12: isocitrate dehydrogenase (IDH); 13: α-oxoglutarate de-hydrogenase (α-KGDH); 14: succinyl-CoA synthetase (LSC); 15: succinate dehydrogenase (SDH); 16: malate dehydrogenase (MDH). Heat map showing gene expression of DEGs under each treatment. Red and green colors indicate up- and down-regulated transcripts, respectively. The column graphs represent changes in metabolites or catalase activity in Medicago sativa leaves. Green and red colors indicate up- and down-regulated transcripts, respectively. Data are means ± SE of three replicates (*n* = 3). Different letters indicate significant differences among treatments (Tukey test at *P* < 0.05)

To further explore NO-involved response mechanisms of alfalfa against PEG stress, 45 DEGs involved in the carbon fixation and TCA cycle metabolic pathways were summarized on a simplified metabolic map (Fig. 4). These DEGs directly or indirectly encode enzymes that catalyze biosynthesis and catabolism. Specifically, from the heatmap, we found genes encode ribulose-bisphosphate carboxylase, glycer-aldehyde-3-phosphate dehydrogenase, fructose-bisphosphate aldolase, and sedoheptulose-bisphosphatase in carbon fixation pathway were significantly down-regulated in PEG-NO concerning those in CK and PEG. In contrast, 7 DEGs encoded aconitate hydra-tase, isocitrate dehydrogenase, and succinyl-CoA synthetase were up-regulated in PEG-NO treatment.

Besides the expression of metabolic enzyme genes, we further determined the key enzyme activities and intermediate metabolites content. Compared with the CK, PEG stress induced a significant increase in FBA, PDH, and CS activity, while a slight increase was observed under PEG-NO treatment. A further novel finding is that the activity of GAPDH, RuBPCase, α-KGDH, and SDH was remarkably decreased under PEG and PEG-NO treatment concerning those of the control, especially in PEG-NO. The NADP-MDH activity under PEG-NO treatment significantly increased relative to that of PEG; there was no noticeable change in IDH activity among the three treatments.

Besides, the citrate acid contents were increased significantly compared to that in CK, whereas there was a decrease in PEG-NO with respect to PEG. Moreover, significant accumulations of amino acids were observed under stress conditions, and the highest content was in the PEG-NO treatment. This is an important finding in understanding the role of endogenous NO in responding to drought stress.

### Hormone-related DEGs and hormone contents

**Fig. 5:**
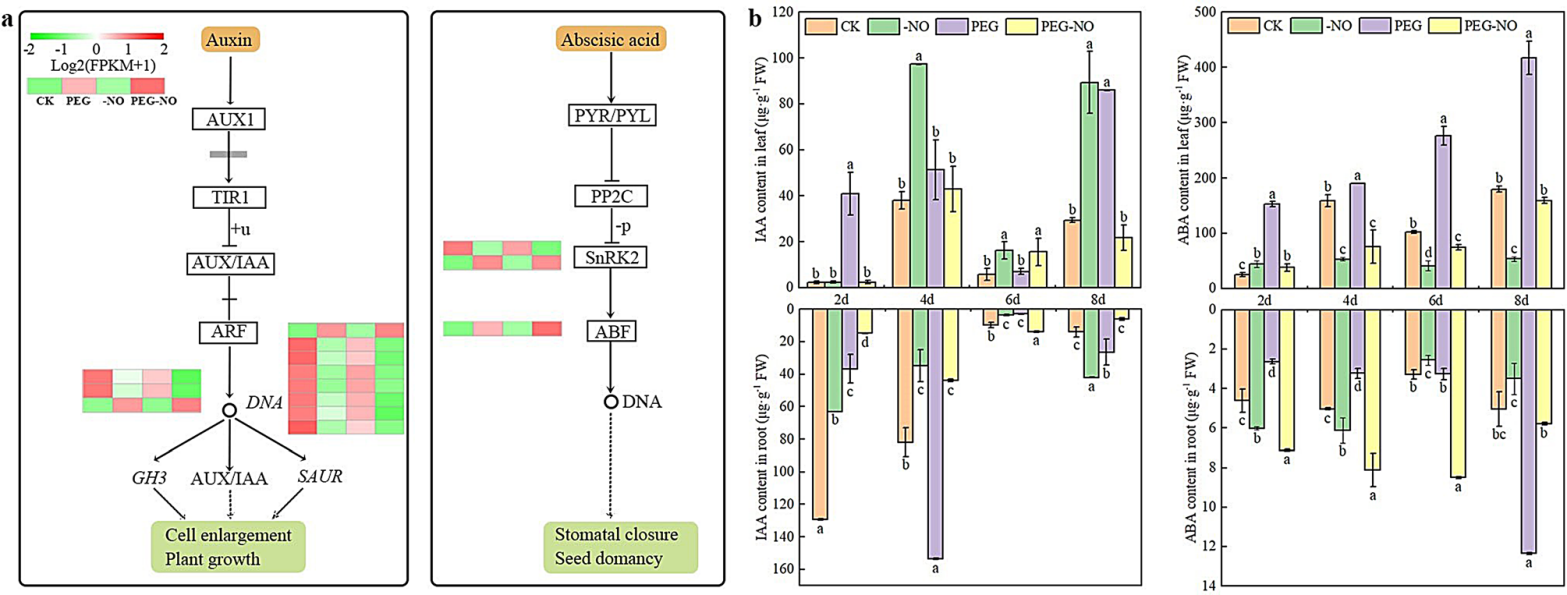
DEGs related to plant hormone signal transduction pathways and endogenous IAA and ABA content involved in *Medicago sativa*. (**a**) Expression profiles of DEGs related to the hormone signal transduction pathways under three treatments. (**b**) Comparison of IAA and ABA concentrations in *Medicago sativa* leaves and roots under three treatments. Data are means ± SE of three replicates (*n* = 3). Different letters indicate significant differences among treatments (Tukey test at *P* < 0.05)

There was a close link between plant hormones and endogenous NO levels; we found 14 genes involved in plant hormone signal transduction pathways (Fig. 5a). For instance, *GH3* and *SAUR* were involved in the auxin signal transduction pathway, and expression levels were significantly down-regulated in PEG-NO compared with those in CK and PEG. The expression levels of one SnRK2 and ABF were significantly up-regulated in PEG-NO compared with those in PEG and CK.

To further investigate the relationship between hormones and endogenous NO, we determined the IAA and ABA contents in alfalfa leaves and roots. As shown in Fig. 5b, the IAA content of alfalfa leaves and roots in each treatment presents a trend of first increasing, then decreasing, and then increasing. In the PEG treatment compared with the CK, IAA content in leaves and roots increased by 1.35, and 1.88-fold respectively on day 4, as well as by 2.92, and 1.90-fold respectively on day 8. However, the application of the NO scavenger inhibited the accumulation of IAA except for day 4. The ABA content in leaves increased continuously over the drought period, but endogenous NO absence declined by 3.99, 2.53, 3.69, and 2.62 times on day 2,4,6,8, respectively. On the contrary, ABA content in roots decreased on days 2 and 4 and increased on day 8 under PEG treatment relative to CK, and yet ABA content significantly increased by 2.71, 2.52, and 2.60 times in PEG-NO compared with PEG at 2, 4, 6 days, respectively (Fig. 5b).

### Validation of transcriptome data by qRT-PCR

**Fig. 6:**
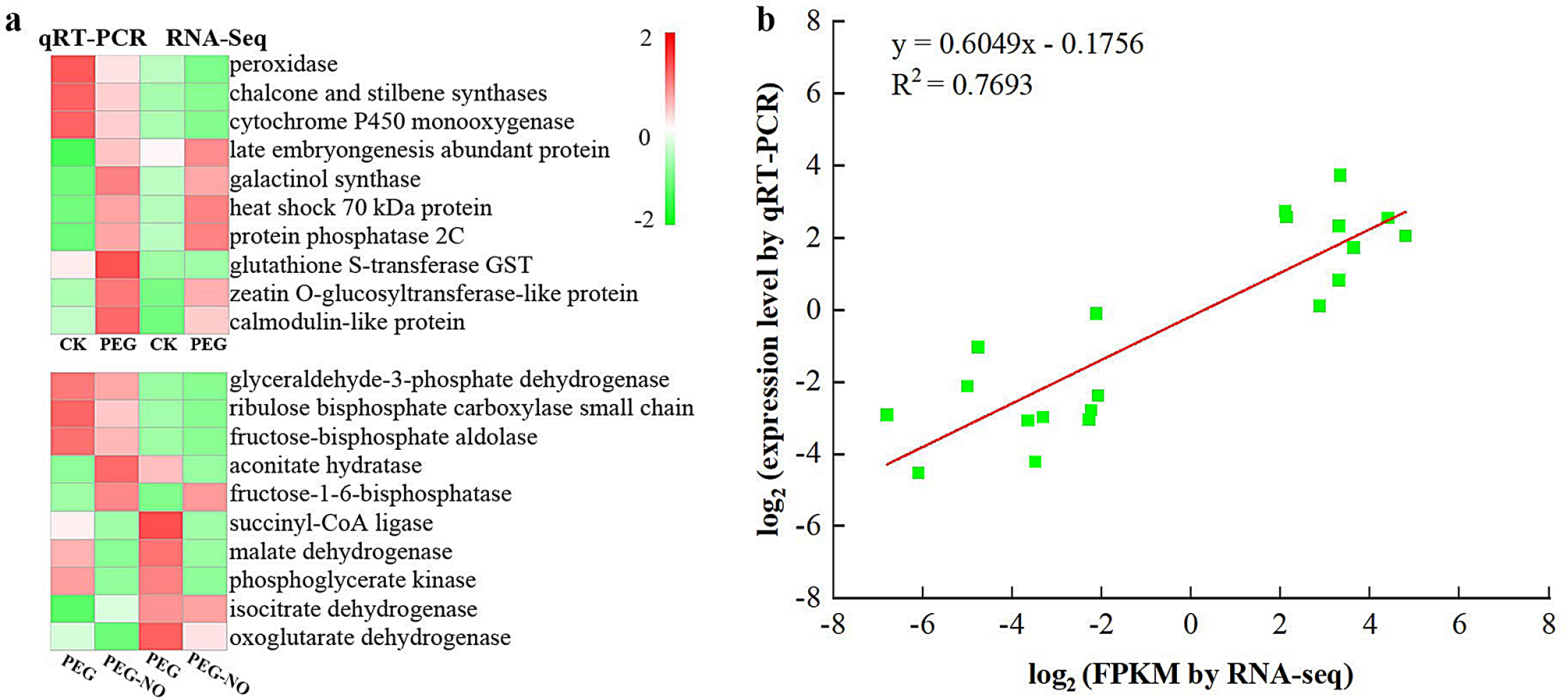
The expression pattern of ten selected genes identified by RNA-Seq was verified by qRT-PCR. (**a**) Heat map showing the expression changes (log2-FPKM) in response to the CK, PEG and PEG-NO treatments for each candidate gene as measured by RNA-Seq and qRT-PCR. (**b**) Scatter plot showing the changes in the expression (log 2-fold change) of selected genes based on RNA-Seq via qRT-PCR. The gene expression levels are indicated by colored bars

To confirm RNA-Seq results, quantitative reverse transcription (qRT-PCR) was conducted on 20 randomly selected DEGs (ten regulated by PEG and ten regulated by NO scavenger) based on transcriptional profile analysis. We further validated the changes in gene expression identified by RNA-Seq by comparing fold changes in sequence reads with fold changes determined by qRT-PCR for each condition. In our analysis, a positive correlation coefficient (R^2^ = 0.7693) was obtained by linear regression analysis, suggesting that the expression of these selected genes in our transcriptome data generally agreed with the qRT-PCR results (Fig. 6, Table S6).

## Discussion

Water deficit severely restricts plant growth and development. However, plants have also established an extensive and complex defense system during the long-term evolutionary process. One of the major signaling molecules present in the cascade of stress adaptation response is nitric oxide (NO), a gaseous and redox-elated signaling molecule [54]. The role of NO in mitigating drought stress effects has been observed in grains, legumes, fruit trees, and vegetables [55]. In this study, we found that the NO content in leaves was consistently higher than the control throughout the PEG stress period. At the same time, NO in roots did not increase significantly until the sixth day of PEG treatment (Fig. 1c, d). The results indicate that alfalfa leaves are more sensitive than roots in responding to water deficiency from the perspective of NO. In addition, endogenous NO has no significant effect on the length of the above-ground stems and the length of the below-ground roots under PEG treatment (Fig. 1b). However, it significantly regulates the branching of stems and the formation of lateral roots (Fig.S1). Plant roots are the first organs to sense water deficit in dehydrating soil and thus play a crucial role in plant drought responses. Liao et al. [56] found that endogenous NO is critical to adventitious root development under drought conditions in marigold explants. This promoting effect was also found in *Cucumis sativus* [57], *Kandelia obovate* [58], and *Lactuca sativa* L [59]. This indicates that endogenous NO-promoted the formation of alfalfa lateral roots can uptake water in the soil more efficiently by increasing the contact area with the soil.

Plant hormones play essential roles in the acclimation of plants to drought stress [60]. Furthermore, the interactions between NO and the plant hormonal pathways also involve drought-stimulated signals [61]. Studies have confirmed root hair, lateral, primary, and adventitious root formation resulted from auxin and NO interact-regulated [62–63]. Auxin stimulates NO generation in soybean roots and cucumber hypocotyls, which was required for adventitious rooting [64–65]. In our study, NO scavenger reduced auxin content in alfalfa roots and leaves under drought stress (Fig. 5b). Auxin early response genes *GH3* and *SAUR* were also down-regulated when NO scavenger was added under drought stress (Fig. 5a). However, the genes that respond to auxin during the germination stage are *AUX1/IAA* and *ARF* [39]. This indicates that alfalfa adapts to water-deficit stress through IAA synthesis and signal transduction mediated by endogenous NO, but the responsive genes vary in different growth stages. *AtGH3-2/YDK1* and *AtGH3-6/DFL1* genes were induced by auxin, and their overexpression lines showed a reduced lateral roots number phenotype [66]. However, in our study, the *GH3* gene positively regulates the lateral roots number of alfalfa (Fig.S1), and the function of the *GH3* gene on alfalfa lateral roots formation needs to be further verified through genetic engineering technology. Excessive expression of *AtSAUR41* led to PR growth and vigorous lateral roots development [67], and *AbSAUR1* overexpressing plants showed increased plant height, thicker stems, and more branches and leaves, besides significantly enlarged and thickened PR, and increased number of lateral roots [68]. This is consistent with our results that high expression of *SAUR* promotes the formation of lateral roots and stem branches (Fig. 5, Fig.S1).

In addition, ABA is also involved in endogenous NO-mediated water deficit stress tolerance to alfalfa. ABA is believed to be a major root-to-shoot inter-organ signal. After plants sense water deficiency, ABA is synthesized in the roots and transported to the shoots via xylem flow [69]. Further, as the enzymes involved in ABA biosynthesis are expressed in leaf vascular, ABA can also accumulate in the vasculature of leaves and then be transported to leaf mesophyll tissues [70–71]. In this study, ABA content in leaves is more than 100 times higher than in roots. NO scavenger significantly inhibited ABA accumulation in the leaves (Fig. 5). This accords with previous observations that NO synthase activity increased after 20 min drought stress and ABA began to accumulate in root tips of wheat (*Triticum aestivum* L.) 60 min later, while pretreated with nitric oxide synthase inhibitor strongly blocked the induction of ABA by drought [72]. Genetic evidence showed that drought stress triggers an increase in ABA concentration, which initiates a signaling cascade to control the activity of anion channels and stomatal aperture, thereby reducing water loss [72]. In the presence of ABA, an ABA molecule binds to ABA receptor PYRABACTIN RESISTANCE/PYR-LIKE (PYR/PYL), and interacts with protein phosphatase 2C (PP2C) then inhibit its activity, promotes sucrose non-fermenting-1-related protein kinase 2 (SnRK2) activate downstream transporters to regulate stomatal responses [70]. At the same time, SnRK2 can respond to ABA and adversity signals by interacting with upstream PP2C proteins and phosphorylating downstream ABF-like transcription factors [73]. Our study revealed endogenous NO scavenging under PEG stress caused the expression level of SnRK2 and ABF genes to decrease (Fig. 5a), and stomatal length, width and aperture were increased significantly (Fig.S2). similarly, prior research indicated that the NO scavenger reversed ABA-induced stomatal [74]. It further supports that NO is crucial for the ABA-regulated signaling pathway that promotes stomatal closure. However, the relationship between the expression levels of *SnRK2* and *AB*F and stomatal opening remains to be further investigated. Moreover, high levels of ABA lead to rapid stomatal closure and reduced photosynthetic capacity, thereby inhibiting the accumulation of carbon-assimilation products and plant growth [75]. Recently, a study reported that SnRK2s repress cellular respiration through the tricarboxylic acid cycle, indicating that SnRK2s have critical roles in energy balance [76].

Carbon metabolism is not only crucial for plant growth and development, but also plays a critical regulatory role in response to adversity. Drought stress has seriously affected the carbon cycling process in plants [77]. In our study, by grouping genes based on biology functions and physiology characteristics, most DGEs regulated by endogenous NO are involved in energy production and conversion (Fig. 2), enriched in carbon fixation in photosynthetic organisms and TCA cycle; meanwhile, more than one-half of DEGs were down-regulated under PEG-NO (Fig.S7, Fig. 4). Especially, compared with our previous study on germination alfalfa sprouts, the genes *Rubisco*, *GAPDH*, *PDH*, *SDH*, and *MDH* were also down-regulated [39], thus, we will further verify these genes functions in future studies. In fact, Meng et al. have provided insights into NO has a distinct influence on carbon fixation and photosynthesis using a label-free proteomics method in cotton leaf [78]. Similarly, Sita et al. argued that heat stress plus exogenous NO significantly improved carbon fixation (as Rubisco activity) and assimilation ability [79]. All evidence supports that NO is involved in plant carbon metabolism. In our study, we found the activity of key enzymes involved in carbon fixation lower in PEG-NO than in PEG treatment (Fig. 4), which implies that endogenous NO deficiency reduces the ability of alfalfa to assimilate carbon. However, PEG-NO leads to a decrease in citric acid content and an increase in amino acid content; we speculate that endogenous NO determines the speed of the TCA cycle. What’s more important, the TCA process generates energy. Previous studies state that using cPTIO leads to a reduction of the ATP/ADP ratio and inhibition of NO production, indicating that NO production is necessary to maintain the energy state [80]. Moreover, NO is an effective inhibitor of cytochrome oxidase in the mitochondrial electron transport chain and has a positive correspondence between NO levels and NADH/NAD and NADPH/NADP ratios in alfalfa root cultures. This is due to low NO levels promoting NAD(P)H turnover through increased malate dehydrogenase activity in alfalfa [81–82]. In our work, the malate dehydrogenase activity was higher in the PEG-NO compared to the PEG treatment, which indicates that endogenous NO plays a crucial role in maintaining cell energy status (ATP levels) during PEG stress. This also indirectly explains that lower concentrations of NO subsequently lead to a lack of required energy which retards the development and growth of the alfalfa plants (Fig.S1).

**Fig. 7:**
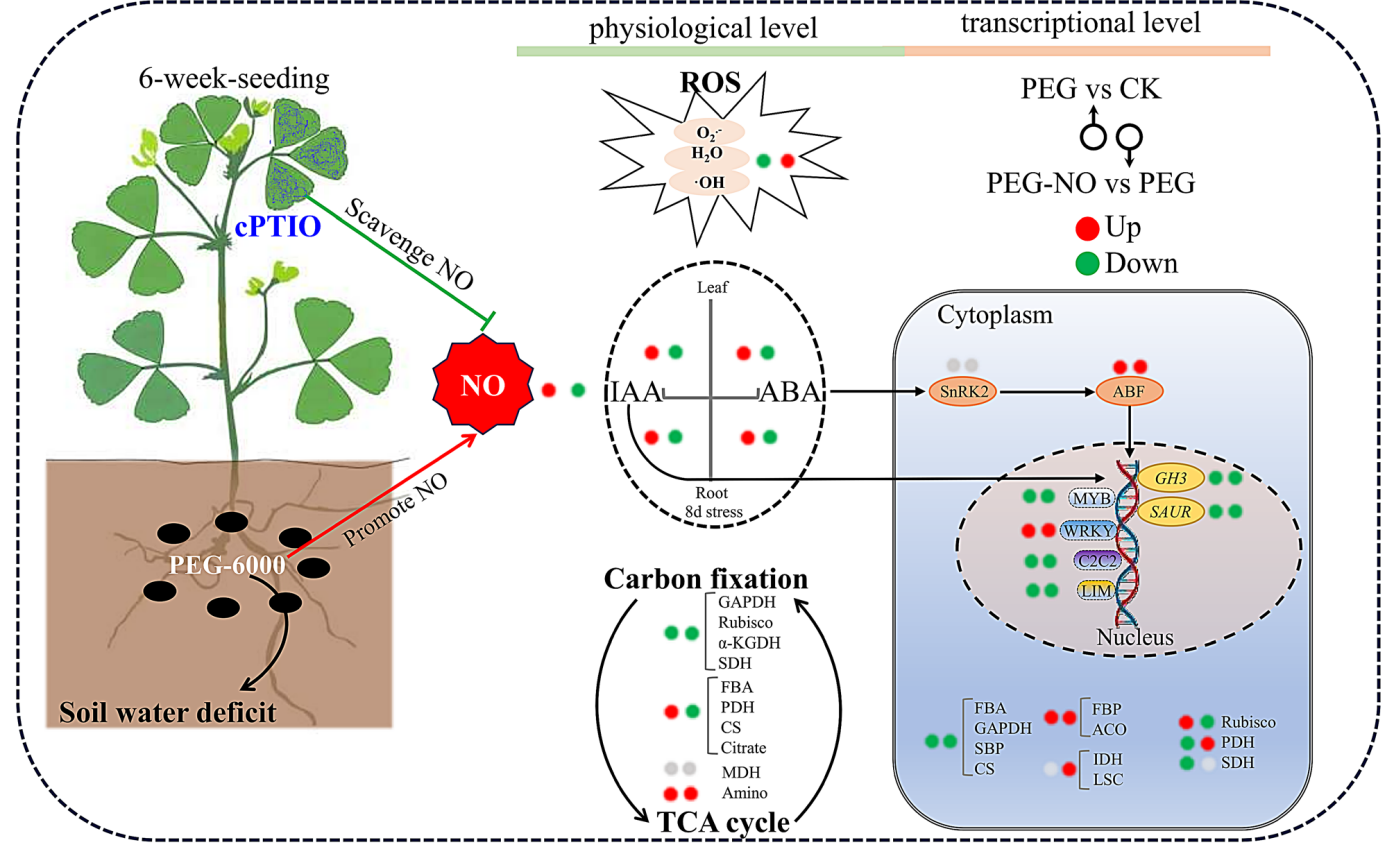
Schematic diagram for the role of endogenous NO in alfalfa under water deficit conditions. Endogenous NO-scavenging exacerbates accumulation of ROS, impedes the accumulation of IAA and ABA, reduces the activity of key enzymes in carbon metabolism. Moreover, Endogenous NO regulates transcription regulators of hormone signaling and many genes involved in carbon metabolism

## Conclusions

In our study, we analyzed the role of endogenous NO in the response of alfalfa to water deficit stress at morphological, physiological, and transcriptional levels. Compared with PEG treatment, PEG-NO inhibits alfalfa’s lateral roots and branch formation, exacerbates H₂O₂, O₂.⁻, and MDA accumulation, and decreases IAA and ABA content. At the the transcriptional level, Carbon fixation, TCA cycle and plant hormones signaling transduction are involved in endogenous NO-mediated water-deficient defense. Genes enriched in these pathways were further analyzed and screened to the *GH3*, *SAUR*, *SnRK2*, *ABF* genes, as well as *FBA*, *GAPDH*, *SBP* and *CS*. These could be used as potential candidate genes for endogenous NO-mediated water-deficit tolerance in alfalfa (Fig. 7). The results of this study provide insights into the mechanisms of drought tolerance in alfalfa and contribute to understanding the physiological role of endogenous NO in drought tolerance in alfalfa.

## Electronic supplementary material

Below is the link to the electronic supplementary material.

Supplementary Material 1

Supplementary Material 2

## Data Availability

The transcriptome sequencing data are available in the National Center for Biotechnology Information (NCBI) SRA repository: PRJNA478630. The datasets were analyzed during this study are included in this published article and its supplementary information files.

## Declarations

### Statement on guidelines

All experimental studies and experimental materials involved in this research are in full compliance with relevant institutional, national, and international guidelines and legislation.

## Abbreviations

cPTIO: 2 - (4-carboxyphenyl) - 4, 4, 5, 5 - tetramethylimidazoline - 1 - oxyl - 3 - oxide potassium
CS: Citrate synthase
DEG: Differentially expressed gene
FBA: Fructose-bisphosphate aldolase
GAPDH: Glyceraldehyde-3-phosphate dehydrogenase
GO: Gene Ontology
IDH: Isocitrate dehydrogenase
KEGG: Kyoto Encyclopedia of Genes and Genomes
MDA: Malondialdehyde
NO: Nitric oxide
PDH: Pyruvate dehydrogenase
PEG: Macrogol
Rubisco: Ribulose-bisphosphate carboxylase
SBP: Sedoheptulose-bisphosphatase
SDH: Succinate dehydrogenase
TF: Transcription factors
α-KGDH: α-oxoglutarate dehydrogenase

## References

[1] Zhang X, Hao Z, Singh VP, Zhang Y, Feng S, Xu Y, Hao F. Drought propagation under global warming: characteristics, approaches, processes, and controlling factors. Sci Total Environ. 2022;838:156021. 35588839 10.1016/j.scitotenv.2022.156021

[2] Gupta A, Rico-Medina A, Caño-Delgado AI. The physiology of plant responses to drought. Volume 368. New York, N.Y.): Science; 2020. pp. 266–9. 10.1126/science.aaz7614 32299946

[3] Huang Z, Liu Y, Cui Z, Fang Y, He H, Liu B, Wu G. Soil water storage deficit of alfalfa (Medicago sativa) grasslands along ages in arid area (China). Field Crop Res. 2018;221:1–6.

[4] Lin S, Medina CA, Boge B, Hu J, Fransen S, Norberg S, Yu LX. Identification of genetic loci associated with forage quality in response to water deficit in autotetraploid alfalfa (*Medicago sativa* L). BMC Plant Biol. 2020;20(1):303. 32611315 10.1186/s12870-020-02520-2 PMC7328273

[5] Baral R, Lollato RP, Bhandari K, Min D. Yield gap analysis of rainfed alfalfa in the united States. Front Plant Sci. 2022;13:931403. 35968131 10.3389/fpls.2022.931403 PMC9363835

[6] Prince S, Anower MR, Motes CM, Hernandez TD, Liao F, Putman L, Mattson R, Seethepalli A, Shah K, Komp M, Mehta P, York LM, Young C, Monteros MJ. Intraspecific variation for leaf physiological and root morphological adaptation to drought stress in alfalfa (*Medicago sativa* L). Front Plant Sci. 2022;13:795011. 35599860 10.3389/fpls.2022.795011 PMC9117100

[7] Zheng G, Fan C, Di S, et al. Over-expression of arabidopsis *EDT1* gene confers drought tolerance in alfalfa (*Medicago sativa* L). Front Plant Sci. 2017;8:2125. 29326737 10.3389/fpls.2017.02125 PMC5733502

[8] Erice G, Louahlia S, Irigoyen JJ, Sanchez-Diaz M, Avice JC. Biomass partitioning, morphology and water status of four alfalfa genotypes submitted to progressive drought and subsequent recovery. J Plant Physiol. 2010;167(2):114–20. 19744745 10.1016/j.jplph.2009.07.016

[9] Ni Y, Guo YJ, Guo YJ, Han L, Tang H, Conyers M. Leaf cuticular waxes and physiological parameters in alfalfa leaves as influenced by drought. Photosynthetica. 2012;50(3):458–66.

[10] Abid M, Haddad M, Khaled AB, Mansour E, Bachar K, Lacheheb B, Ferchichi A. Water relations and gas exchange in alfalfa leaves under drought conditions in *Southern Tunisian oases*. Pol J Environ Stud. 2016;25:917–24.

[11] Erice G, Louahlia S, Irigoyen JJ, Sánchez-Díaz M, Alami IT, Avice J. Water use efficiency, transpiration and net CO_2_ exchange of four alfalfa genotypes submitted to progressive drought and subsequent recovery. Environ Exp Bot. 2011;72:123–30.

[12] Irigoyen JJ, Emerich DW, Sánchez-Díaz M. Alfalfa leaf senescence induced by drought stress: photosynthesis, hydrogen peroxide metabolism, lipid peroxidation and ethylene evolution. Physiol Plant. 2006;84(1):67–72.

[13] Zhang C, Shi S, Wang B, Zhao J. Physiological and biochemical changes in different drought-tolerant alfalfa (*Medicago sativa* L.) varieties under PEG-induced drought stress. Acta Physiol Plant. 2018; 40.

[14] Schubert S, Serraj R, Plies-Balzer E, et al. Effect of drought stress on growth, sugar concentrations and amino acid accumulation in N2-fixing alfalfa (*Medicago sativa* L). J Plant Physiol. 1995;146(4):541–6.

[15] Naya L, Ladrera R, Ramos J, et al. The response of carbon metabolism and antioxidant defenses of alfalfa nodules to drought stress and to the subsequent recovery of plants. Plant Physiol. 2007;144(2):1104–14. 17468213 10.1104/pp.107.099648 PMC1914205

[16] Demirkol G. *PopW* enhances drought stress tolerance of alfalfa via activating antioxidative enzymes, endogenous hormones, drought related genes and inhibiting senescence genes. Plant Physiol Bioch. 2021;166:540–8. 10.1016/j.plaphy.2021.06.036 34174659

[17] Soba D, Zhou B, Arrese-Igor C, Munné-Bosch S, Aranjuelo I, Physiological. Hormonal and metabolic responses of two alfalfa cultivars with contrasting responses to drought. Int J Mol Sci. 2019;20(20):5099. 31618819 10.3390/ijms20205099 PMC6829892

[18] Ma Q, Xu X, Xie Y, Huang T, Wang W, Zhao L, Ma D. Comparative metabolomic analysis of the metabolism pathways under drought stress in alfalfa leaves. Environ Exp Bot. 2021;183:104329.

[19] Xu J, Li XL, Luo L..Effects of engineered Sinorhizobium meliloti on cytokinin synthesis and tolerance of alfalfa to extreme drought Stress [J]. Appl Environ Microbiol. 2012;78(22):8056–61. 22961897 10.1128/AEM.01276-12 PMC3485951

[20] Shi K, Liu J, Liang H, Dong H, Zhang J, Wei Y, Zhou L, Wang S, Zhu J, Cao M, Jones CS, Ma D, Wang Z. An alfalfa MYB-like transcriptional factor MsMYBH positively regulates alfalfa seedling drought resistance and undergoes MsWAV3-mediated degradation. J Integr Plant Biol. 2024;66(4):683–99. 38358036 10.1111/jipb.13626

[21] Rezayian M, Ebrahimzadeh H. Metabolic and physiological changes induced by nitric oxide and its impact on drought tolerance in soybean. J Plant Growth Regul. 2023;42:1905–18.

[22] Ma Q, Xu X, Wang W, Zhao L, Ma D, Xie Y. Comparative analysis of alfalfa (*Medicago sativa* L.) seedling transcriptomes reveals genotype-specific drought tolerance mechanisms. Plant Physiol Biochem. 2021;166:203–14. 34118683 10.1016/j.plaphy.2021.05.008

[23] Du W, Yang J, Li Q, Jiang W, Pang Y. Medicago truncatula β-glucosidase 17 contributes to drought and salt tolerance through antioxidant flavonoid accumulation. Plant Cell Environ. 2024;47(8):3076–89. 38679945 10.1111/pce.14928

[24] Shi K, Liu J, Liang H, Dong H, Zhang J, Wei Y, Zhou L, Wang S, Zhu J, Cao M, Jones CS, Ma D, Wang Z. An alfalfa MYB-like transcriptional factor MsMYBH positively regulates alfalfa seedling drought resistance and undergoes MsWAV3-mediated degradation. J Integr Plant Biol. 2024;66(4):683–99. 10.1111/jipb.13626. 38358036 10.1111/jipb.13626

[25] Luo D, Liu J, Wu Y, Zhang X, Zhou Q, Fang L, Liu Z. NUCLEAR TRANSPORT FACTOR 2-LIKE improves drought tolerance by modulating leaf water loss in alfalfa (*Medicago sativa* L). Plant J. 2022;112(2):429–50. 36006043 10.1111/tpj.15955

[26] Luo S, Liu J, Shi K, Zhang J, Wang Z. Integrated transcriptomic and metabolomic analyses reveal that *MsSPHK1* - A sphingosine kinase gene negatively regulates drought tolerance in alfalfa (*Medicago sativa* L). Plant Physiol Biochem. 2025;218:109302. 39579717 10.1016/j.plaphy.2024.109302

[27] Qiao W, Fan L. Nitric oxide signaling in plant responses to abiotic stresses. J Integr Plant Biol. 2008;50:1238–46. 19017111 10.1111/j.1744-7909.2008.00759.x

[28] Santisree P, Bhatnagar-Mathur P, Sharma KK. NO to drought-multifunctional role of nitric oxide in plant drought: do we have all the answers? Plant Sci. 2015;239:44–55. 26398790 10.1016/j.plantsci.2015.07.012

[29] Yu M, Lamattina L, Spoel SH, Loake GJ. Nitric oxide function in plant biology: a redox cue in Deconvolution. New Phytol. 2014;202:1142–56. 24611485 10.1111/nph.12739

[30] Li Y, Wu Y, Liao W, Hu L, Dawuda MM, Jin X, Tang Z, Yang J, Yu J. Nitric oxide is involved in the brassinolide-induced adventitious root development in cucumber. BMC Plant Biol. 2020; 20. 10.1186/s12870-020-2320-y PMC7059714 32138654

[31] Terrón-Camero LC, Peláez-Vico MÁ, Del-Val C, Sandalio LM, Romero-Puertas MC. Role of nitric oxide in plant responses to heavy metal stress: exogenous application versus endogenous production. J Exp Bot. 2019;70:4477–88. 31125416 10.1093/jxb/erz184

[32] Gan L, Wu X, Zhong Y. Exogenously applied nitric oxide enhances the drought tolerance in hulless barley. Plant Prod Sci. 2015;18:52–6. 10.1626/pps.18.52.

[33] Rezayian M, Ebrahimzadeh H, Niknam V. Metabolic and physiological changes induced by nitric oxide and its impact on drought tolerance in soybean. J Plant Growth Regul. 2022;42:1905–18.

[34] Rigui AP. Victória.Carvalho, André Luiz.Wendt Dos Santos. Fructan and antioxidant metabolisms in plants of Lolium perenne under drought are modulated by exogenous nitric oxide. Plant Physiol Biochem. 2019;145:205–15. 31707248 10.1016/j.plaphy.2019.10.029

[35] Montilla-Bascón G, Rubiales D, Hebelstrup KH, et al. Reduced nitric oxide levels during drought stress promote drought tolerance in barley and is associated with elevated polyamine biosynthesis. Sci Rep. 2017;7:13311. 29042616 10.1038/s41598-017-13458-1 PMC5645388

[36] Wang X, Ruan M, Wan Q, He W, Yang L, Liu X, He L, Yan L, Bi Y. Nitric oxide and hydrogen peroxide increase glucose-6-phosphate dehydrogenase activities and expression upon drought stress in soybean roots. Plant Cell Rep. 2020;39(1):63–73. 31535176 10.1007/s00299-019-02473-3

[37] Singh N, Bhatla SC. Nitric oxide and iron modulate Heme Oxygenase activity as a long distance signaling response to salt stress in sunflower seedling cotyledons. Nitric Oxide-Biol Ch. 2016;53:54–64. 10.1016/j.niox.2016.01.003 26778276

[38] Arasimowicz Jelonek M, Floryszak Wieczorek J, Kosmala A. Are nitric oxide donors a valuable tool to study the functional role of nitric oxide in plant metabolism? Plant Biol. 2011;13:747–56. 21815979 10.1111/j.1438-8677.2010.00430.x

[39] Zhao Y, Wei X, Ji X, Ma W. Endogenous NO-mediated transcripts involved in photosynthesis and carbohydrate metabolism in alfalfa (*Medicago sativa* L.) seedlings under drought stress. Plant Physiol Bioch. 2019;141:456–65. 10.1016/j.plaphy.2019.06.023 31247428

[40] Zhao Y, Wei X, Long Y, Ji X. Transcriptional analysis reveals sodium Nitroprusside affects alfalfa in response to PEG-induced osmotic stress at germination stage. Protoplasma. 2020;257:1345–58. 32556557 10.1007/s00709-020-01508-x

[41] Zhao Y, Ma W, Wei X, Long Y, Zhao Y, Su M, Luo Q. Identification of exogenous nitric Oxide-Responsive MiRNAs from alfalfa (*Medicago sativa* L.) under drought stress by High-Throughput sequencing. Genes. 2020;11:30. 10.3390/genes11010030. 10.3390/genes11010030 PMC7016817 31888061

[42] Ruan Q, Bai X, Wang Y, Zhang X, Wang B, Zhao Y, Zhu X, Wei X. Regulation of endogenous hormone and miRNA in leaves of alfalfa (*Medicago sativa* L.) seedlings under drought stress by endogenous nitric oxide. BMC Genomics. 2024; 1;25(1):229. 10.1186/s12864-024-10024-8 PMC10908014 38429670

[43] Wei B, Wang Y, Ruan Q, Zhu X, Wang X, Wang T, Zhao Y, Wei X. Mechanism of action of microRNA166 on nitric oxide in alfalfa (Medicago sativa L.) under drought stress. BMC Genomics. 2024;25(1):316. 38549050 10.1186/s12864-024-10095-7 PMC10976769

[44] Grabherr MG, Haas BJ, Yassour M, et al. Full-length transcriptome assembly from RNA-Seq data without a reference genome. Nat Biotechnol. 2011;29(7):644–52. 21572440 10.1038/nbt.1883 PMC3571712

[45] Altschul SF, Madden TL, Schäffer AA, et al. Gapped BLAST and PSI-BLAST: a new generation of protein database search programs. Nucleic Acids Res. 1997;25:3389–402. 9254694 10.1093/nar/25.17.3389 PMC146917

[46] Eddy SR. Profile hidden Markov models. Bioinformatics. 1998;14:755–63. 9918945 10.1093/bioinformatics/14.9.755

[47] Ander S, Huber W. Differential expression analysis for sequence count data. Genome Biol. 2010;11:R101–6. 20979621 10.1186/gb-2010-11-10-r106 PMC3218662

[48] Kang Y, Seminario A, Udvardi M, Annicchiarico P. Physiological and biochemical adaptive traits support the specific breeding of alfalfa (*Medicago sativa*) for severely drought-stressed or moisture-favourable environments. J Agron Crop Sci. 2023;209:132–43.

[49] Livak KJ, Schmittgen TD. Analysis of relative gene expression data using real-time quantitative PCR and the 2^– ∆∆CT^ method. Methods. 2001;25:402–8. 11846609 10.1006/meth.2001.1262

[50] Peever TL, Higgins VJ. Electrolyte leakage, Lipoxygenase, and lipid peroxidation induced in tomato leaf tissue by specific and nonspecific elicitors from *Cladosporium fulvum*. Plant Physiol. 1989;90(3):867–75. 10.1104/pp.90.3.867 PMC1061813 16666890

[51] Elstner EF, Heupel A. Inhibition of nitrite formation from hydroxylammoniumchloride: a simple assay for superoxide dismutase. Anal Biochem. 1976;70:616–20. 817618 10.1016/0003-2697(76)90488-7

[52] Loreto F, Velikova V. Isoprene produced by leaves protects the photosynthetic apparatus against Ozone damage, quenches Ozone products, and reduces lipid peroxidation of cellular membranes. Plant Physiol. 2001;127:1781–7. 11743121 PMC133581

[53] Qi N, Hou X, Wang C, Li C, Huang D, Li Y, Wang N, Liao W. Methane-rich water induces bulblet formation of scale cuttings in Lilium Davidii Var. Unicolor by regulating the signal transduction of phytohormones and their levels. Physiol Plant. 2021;172:1919–30. 33748992 10.1111/ppl.13401

[54] Kaya C, Ashraf M, Alyemeni MN, Ahmad P. Responses of nitric oxide and hydrogen sulfide in regulating oxidative defense system in wheat plants grown under cadmium stress. Physiol Plant. 2020;168:345–60. 31343742 10.1111/ppl.13012

[55] Lau S, Hamdan MF, Pua T, Saidi NB, Tan BC. Plant nitric oxide signaling under drought stress. Plants. 2021;10(2):360. 33668545 10.3390/plants10020360 PMC7917642

[56] Liao W, Huang G, Yu J, Zhang M. Nitric oxide and hydrogen peroxide alleviate drought stress in marigold explants and promote its adventitious root development. Plant Physiol Bioch. 2012;58:6–15. 10.1016/j.plaphy.2012.06.012 22771430

[57] Li S, Wan L, Nie Z, Li X. Fractal and topological analyses and antioxidant defense systems of alfalfa (Medicago sativa L.) root system under drought and rehydration regimes. Agronomy. 2020;10:805.

[58] Wei M, Li H, Zhong Y, Shen Z, Ma D, Gao C, Liu Y, Wang W, Zhang J, You Y, Zheng H. Transcriptomic analyses reveal the effect of nitric oxide on the lateral root development and growth of Mangrove plant Kandelia obovata. Plant Soil. 2022;472:543–64.

[59] Lombardo MC, Graziano M, Polacco JC, Lamattina L. Nitric oxide functions as a positive regulator of root hair development. Plant Signal Behav. 2006;1:28–33. 19521473 10.4161/psb.1.1.2398 PMC2633697

[60] Waadt R, Seller CA, Hsu PK, Takahashi Y, Munemasa S, Schroeder JI. Plant hormone regulation of abiotic stress responses. Nat Rev Mol Cell Bio. 2022;23. 10.1038/s41580-022-00479-6 PMC9592120 35513717

[61] Prakash V, Singh VP, Tripathi DK, Sharma S, Corpas FJ. Crosstalk between nitric oxide (NO) and abscisic acid (ABA) signalling molecules in higher plants. Environ Exp Bot; 2018.

[62] Correa-Aragunde N, Graziano M, Lamattina L. Nitric oxide plays a central role in determining lateral root development in tomato. Planta. 2004;218:900–5. 14716561 10.1007/s00425-003-1172-7

[63] Reeksting BJ, Olivier NA, van den Berg N. Transcriptome responses of an ungrafted Phytophthora root rot tolerant avocado (*Persea americana*) rootstock to flooding and *Phytophthora cinnamomi*. Bmc Plant Biol. 2016; 16. 10.1186/s12870-016-0893-2 PMC5034587 27658453

[64] Hu XY, Neill SJ, Tang ZC, Cai WM. Nitric oxide mediates gravitropic bending in soybean roots. Plant Physiol. 2005;137:663–70. 15681661 10.1104/pp.104.054494 PMC1065366

[65] Pagnussat GC, Simontacchi M, Puntarulo S, Lamattina L. Nitric oxide is required for root organogenesis. Plant Physiol. 2002;129:954–6. 12114551 10.1104/pp.004036 PMC1540240

[66] Nakazawa M, Yabe N, Ichikawa T, et al. DFL1, an auxin-responsive GH3 gene homologue, negatively regulates shoot cell elongation and lateral root formation and positively regulates the light response of hypocotyl length. Plant J. 2010;25(2):213–21. 10.1046/j.1365-313x.2001.00957.x 11169197

[67] Qiu T, Chen Y, Li M, et al. The tissue-specific and developmentally regulated expression patterns of the SAUR41 subfamily of SMALL AUXIN UP RNA genes. Plant Signal Behav. 2014;8(8):e25283. 10.4161/psb.25283 PMC3999058 23759547

[68] Bai F, Li S, Yang C, Zhao T, Zhang T, Lan X. Overexpression of the *AbSAUR1* gene enhanced biomass production and alkaloid yield in Atropa belladonna. Ind Crops Prod. 2019;140:111705.

[69] Christmann A, Grill E, Huang J. Hydraulic signals in long-distance signaling. Curr Opin Plant Biol. 2013:16. 10.1016/j.pbi.2013.02.011 23545219

[70] Chen K, Li GJ, Bressan RA, Song CP, Zhu JK, Zhao Y. Abscisic acid dynamics, signaling, and functions in plants. J Integr Plant Biol. 2020;62:25–54. 31850654 10.1111/jipb.12899

[71] Hsu P, Dubeaux G, Takahashi Y, Schroeder JI. Signaling mechanisms in abscisic acid-mediated stomatal closure. Plant J. 2021;105:307–21. 33145840 10.1111/tpj.15067 PMC7902384

[72] Munemasa S, Hauser F, Park J, Waadt R, Brandt B, Schroeder JI. Mechanisms of abscisic acid-mediated control of stomatal aperture. Curr Opin Plant Biol. 2015;28:154–62. 26599955 10.1016/j.pbi.2015.10.010 PMC4679528

[73] Fujita Y, Yoshida T, Yamaguchi-Shinozaki K. Pivotal role of the AREB/ABF-SnRK2 pathway in ABRE-mediated transcription in response to osmotic stress in plants. Physiol Plant. 2013;147:15–27. 22519646 10.1111/j.1399-3054.2012.01635.x

[74] Sami F, Faizan M, Faraz A, Siddiqui H, Yusuf M, Hayat S. Nitric oxide-mediated integrative alterations in plant metabolism to confer abiotic stress tolerance, NO crosstalk with phytohormones and NO-mediated post translational modifications in modulating diverse plant stress. Nitric Oxide. 2018;73:22–38. 29275195 10.1016/j.niox.2017.12.005

[75] Antoniou C, Xenofontos R, Chatzimichail G, Christou A, Kashfi K, Fotopoulos V. Exploring the potential of nitric oxide and hydrogen sulfide (NOSH)-Releasing synthetic compounds as novel priming agents against drought stress in *Medicago sativa* plants. Biomolecules. 2020;10:120. 31936819 10.3390/biom10010120 PMC7023404

[76] Yoshida T, Obata T, Feil R, Lunn JE, Fujita Y, Yamaguchi-Shinozaki K, Fernie AR. The role of abscisic acid signaling in maintaining the metabolic balance required for Arabidopsis growth under nonstress conditions. Plant Cell. 2019;31:84–105. 30606780 10.1105/tpc.18.00766 PMC6391705

[77] Li L, Li Y, Ding G. Response mechanism of carbon metabolism of Pinus massoniana to gradient high temperature and drought stress. BMC Genomics. 2024;25(1):166. 38347506 10.1186/s12864-024-10054-2 PMC10860282

[78] Meng Y, Liu F, Pang C, Fan S, Song M, Wang D, Li W, Yu S. Label-free quantitative proteomics analysis of cotton leaf response to nitric oxide. J Proteome Res. 2011;10:5416–32. 22029526 10.1021/pr200671d

[79] Sita K, Sehgal A, Bhardwaj A, Bhandari K, Kumar S, Prasad PV, Jha U, Siddique KHM, Nayyar H. Nitric oxide secures reproductive efficiency in heat-stressed lentil (*Lens culinaris Medik*.) plants by enhancing the photosynthetic ability to improve yield traits. Physiol Mol Biol Pla. 2021;27:2549–66. 10.1007/s12298-021-01098-9 PMC8639968 34924710

[80] Aridhi F, Sghaier H, Gaitanaros A, Khadri A, Aschi-Smiti S, Brouquisse R. Nitric oxide production is involved in maintaining energy state in alfalfa (*Medicago sativa* L.) nodulated roots under both salinity and flooding. Planta. 2020; 252. 10.1007/s00425-020-03422-1 32676756

[81] Zottini M, Formentin E, Scattolin M, Carimi F, Lo Schiavo F, Terzi M. Nitric oxide affects plant mitochondrial functionality in vivo. FEBS Lett. 2002;515(1–3):75–8. 11943198 10.1016/s0014-5793(02)02438-9

[82] Igamberdiev AU, Sereg Lyes C, Manac’H N, Hill RD. NADH-dependent metabolism of nitric oxide in alfalfa root cultures expressing barley hemoglobin. Planta. 2004;219:95–102. 14740214 10.1007/s00425-003-1192-3

