# Supplementary material for: RNA-Seq uncovers endogenous NO-induced hormone signal transduction and carbon metabolism in response to PEG stress in alfalfa
Source: BMC Genomics. 2025 May 23;26:523. doi: 10.1186/s12864-025-11706-7 (PMC12101008; doi:10.1186/s12864-025-11706-7)
Supplement: Supplementary file 1 — Supplementary Material 1 [file 12864_2025_11706_MOESM1_ESM.docx]

**Supplementary Figure**


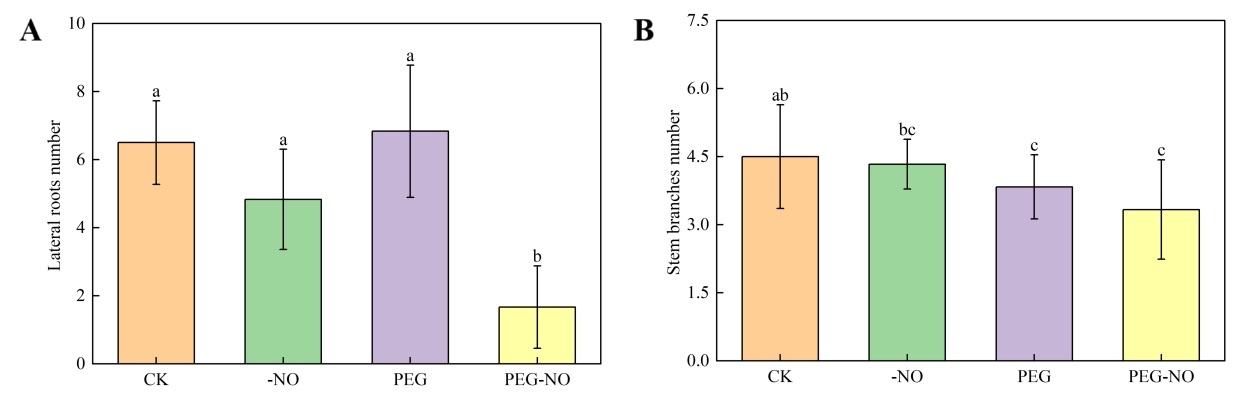
**Fig. S1** Effects of endogenous NO on stem branches number, and lateral roots number of alfalfa seedlings under PEG stress.

**
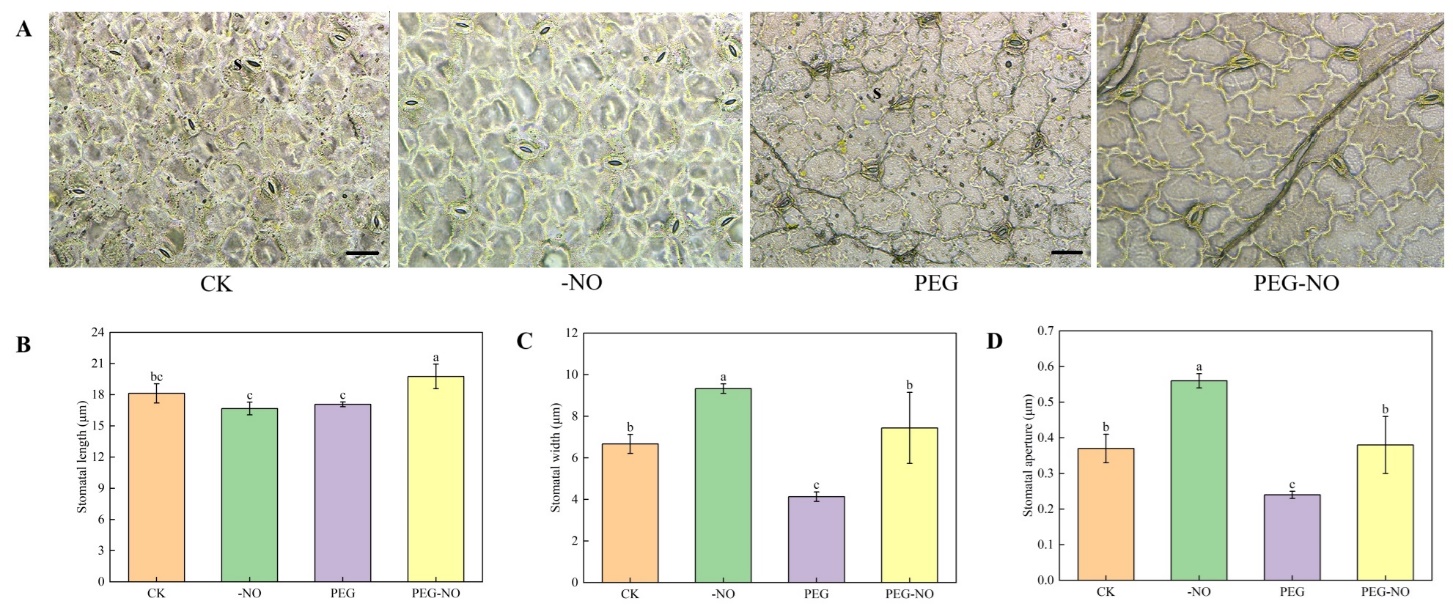
Fig. S2** Stomatal characteristics of alfalfa leaves under PEG and NO scavenger treatment.


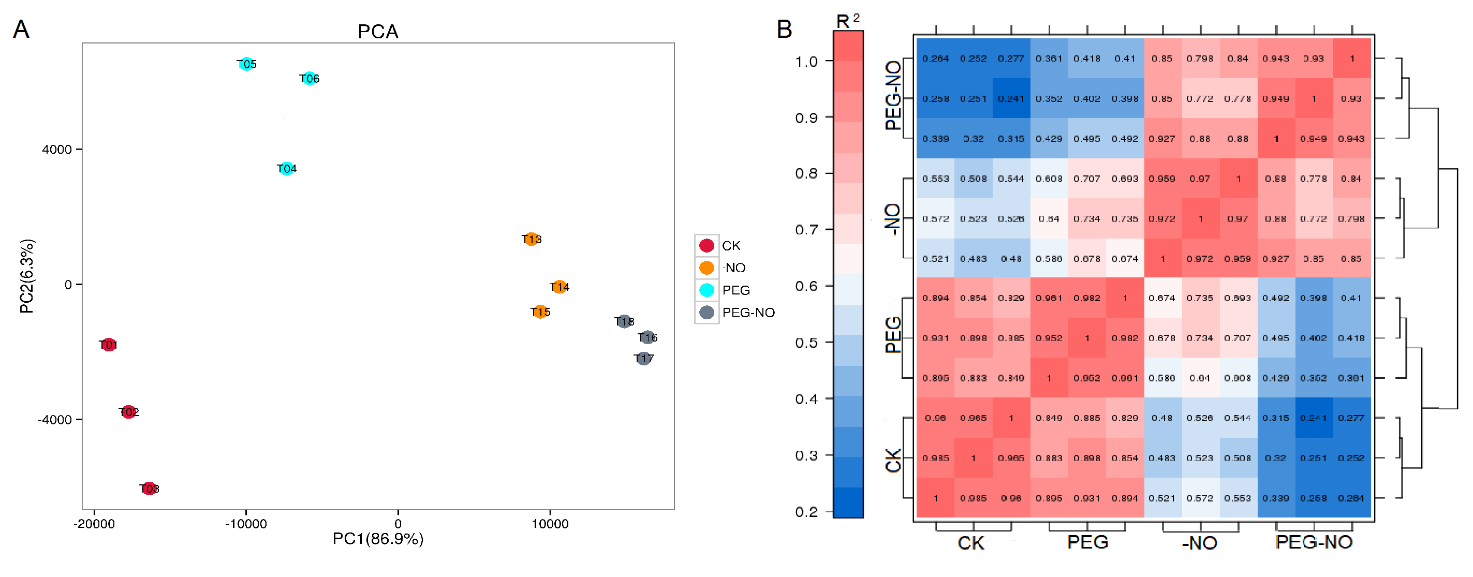


**Fig. S3** Statistical analysis of sequencing data. **A** Principal component analysis of all samples, **B** Pearson’s

correlation coefficients among samples.


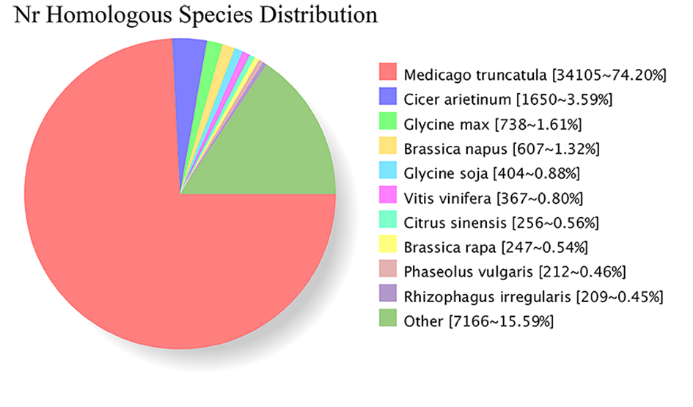


**Fig. S4** The species distribution of unigene blast results against the NR protein database.


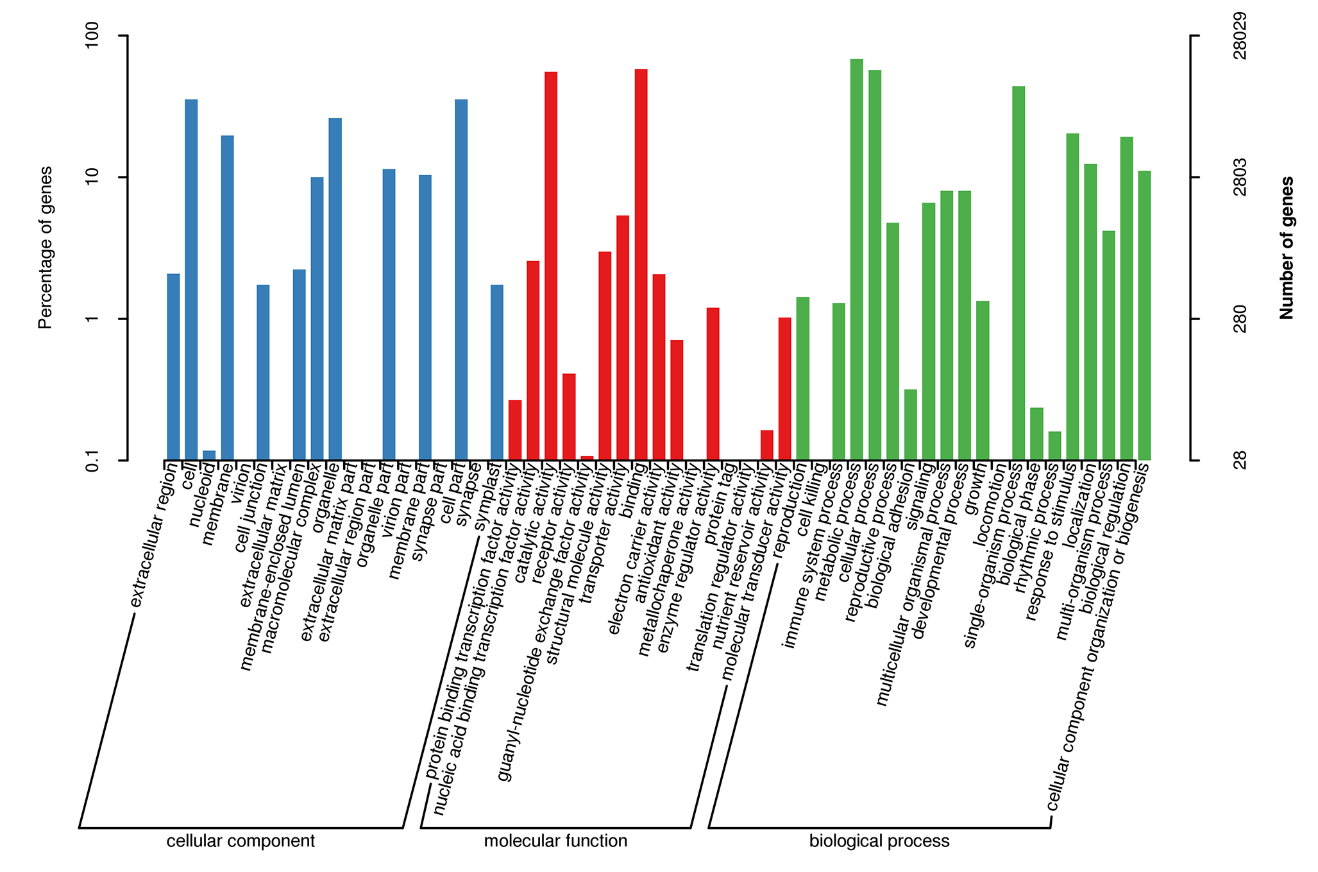


**Fig. S5** Gene ontology classification of assembled unigenes of *M. sativa*. Results are summarized in three categories: cellular component, molecular function, and biological process. The left x-axis indicates the percentage of a specific category of genes in that main category. The right x-axis indicates the actual number of genes in a category.

**
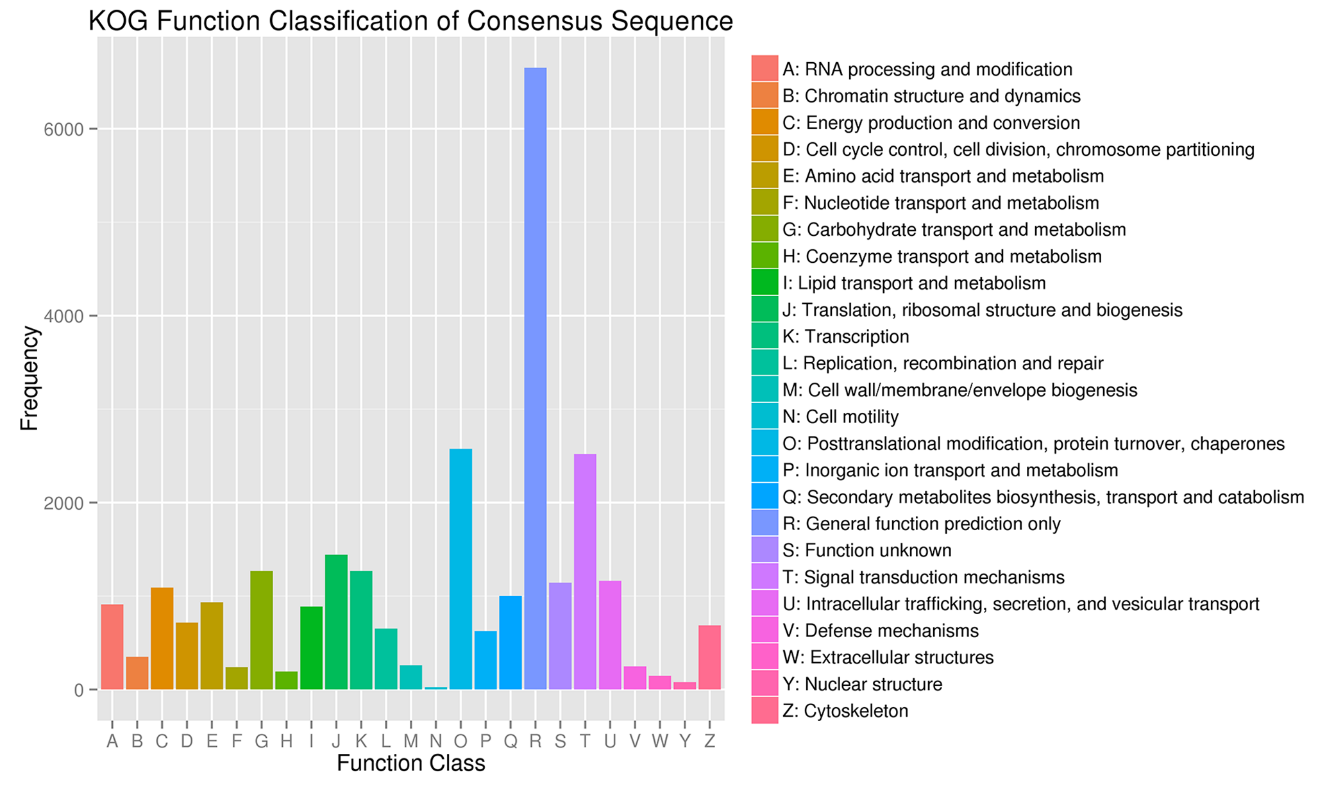
Fig. S6** Histogram presentation of KOG classification of *M. sativa* unigenes. A total of 24,483 sequences have a KOG classification among the 25 categories.


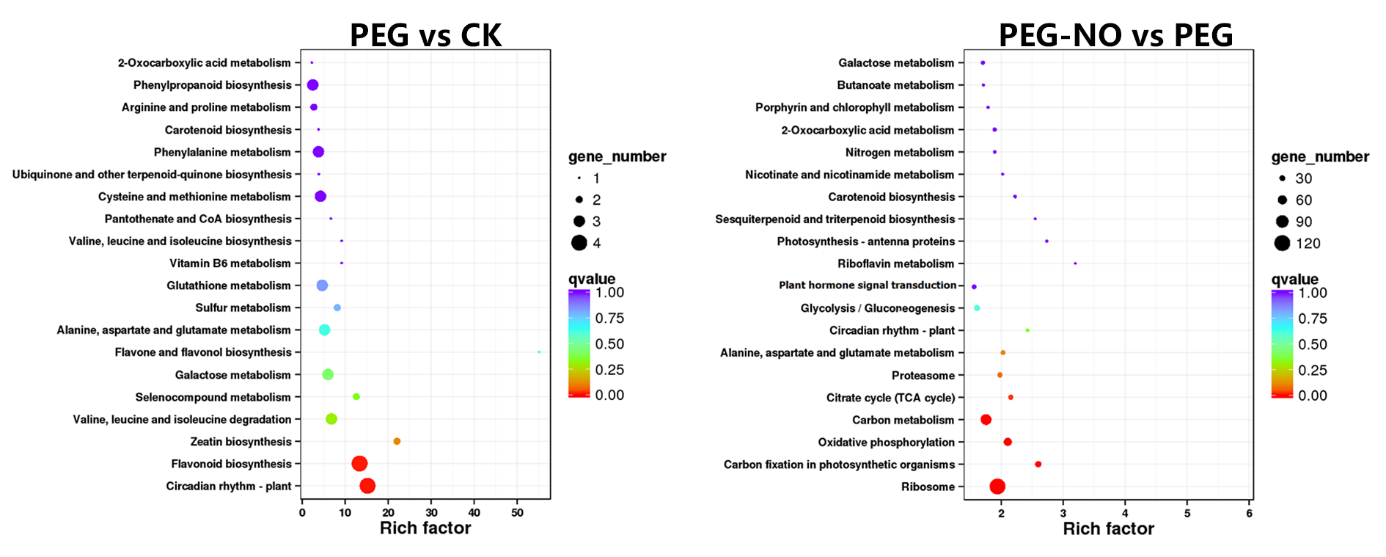


**Fig. S7** Scatterplot of enriched KEGG pathways for differentially expressed genes under PEG and mannitol stress. The rich factor is the ratio of the DEG number to the total gene number in a particular pathway. The size and color of the dots represent the gene number and the range of the -log10 (q-value), respectively.
